# Directed Dynamic Resolution of an Atropisomeric Silver Complex in Solution: Role of π–π Interactions

**DOI:** 10.1021/acs.inorgchem.5c03548

**Published:** 2025-11-05

**Authors:** Alvaro Polo, Ricardo Rodríguez, Pablo J. Sanz Miguel

**Affiliations:** Departamento de Química Inorgánica, 16765 Instituto de Síntesis Química y Catálisis Homogénea (ISQCH), Universidad de Zaragoza-CSIC, 50009 Zaragoza, Spain

## Abstract

We report a directed dynamic resolution process for an atropisomeric [Ag­(NHC)_2_]^+^ complex, where stereochemical control is achieved through enantiomeric interconversion in solution before crystallization. Unlike conventional diastereomeric salt formation, our approach shifts the restricted interconversion between *R*_a_ and *S*_a_ atropisomers, allowing selective stabilization of one enantiomer via π–π interactions with enantiopure binaphthalene-based borate anions in solution. NMR spectroscopy reveals conformational locking and restricted dynamics preceding crystallization, which captures the resolved stereochemistry in highly crystalline salts. This study demonstrates that noncovalent interactions effectively modulate molecular conformational dynamics to achieve chiral resolution.

## Graphical abstract

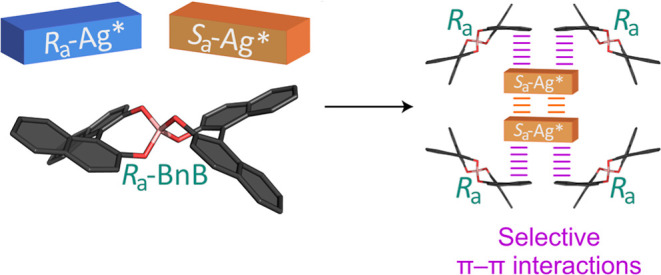

## Introduction

Resolution of racemic mixtures into their individual enantiomers has been achieved through a variety of methodologies.^1^ A particularly elegant approach is preferential crystallization.^2,3^ In this method, a supersaturated racemic solution (A + B) is seeded with crystals of a single enantiomer (e.g., A) in order to promote selective nucleation and growth of that enantiomer (A), while leaving the other enantiomer (B) in solution. At a more fundamental level, spontaneous resolution deserves special mention. This process, which enabled Pasteur to postulate the concept of molecular chirality,^4^ does not require any external chiral sources. Instead, it typically operates under supramolecular control, being highly influenced by stereospecific noncovalent interactions such as hydrogen bonding, π–π stacking, and van der Waals forces.^5–7^ Additional resolution strategies include enzymatic and chemical approaches, which exploit the differential reactivity of the enantiomers involved.^8^ These methods selectively convert one enantiomer into a different compound, leaving the other unaltered. Similarly, chromatographic techniques, as chiral high-performance liquid chromatography (HPLC) or supercritical fluid chromatography (SFC), utilize chiral stationary phases or chiral additives to achieve enantioseparation with high efficiency.^9–11^

Among the various approaches, diastereomeric salt formation is probably the most widely used.^12,13^ In this process, a racemic mixture (A + B) is typically reacted with a chiral auxiliary (C*), leading to the formation of two diastereomers (AC* + BC*). Due to their different physicochemical properties, particularly solubility, one diastereomer can be selectively isolated and subsequently converted to the desired enantiomer. Typical resolving agents include chiral pool scaffolds, such as amino acid derivatives, and enantiopure anions.^1,14^

Resolution of saturated cationic asymmetric metal complexes can be efficiently achieved through the use of chiral anions, which induce diastereoselectivity via ion-pair formation. When sterogenically labile complex cations interact with enantiopure additives, such as chiral anions, the racemic equilibrium can shift toward the preferential formation of an enantioenriched cation, or even a single enantiomer cation, via the assembly of supramolecular ion pairs. This phenomenon, commonly referred to as the Pfeiffer effect,^15^ was first postulated by Pfeiffer in the 1930s while studying the enhanced optical rotation of optically active alkaloids in the presence of labile racemic transition-metal complexes. The effect arises from the formation of diastereomeric ion pairs, which can induce significant levels of chiral recognition. Lacour pioneered the application of this concept by demonstrating the diastereoselective formation of ion pairs in solution,^16,17^ demonstrating the ability of chiral anions to promote the formation of a single diastereoisomer of salts of conformationally labile chiral octahedral cations.^18,19^ The efficiency of the chiral recognition process arises from subtle differences in electrostatic interactions between cation–anion pairs.^20^ Thus, selective ion pair formation is highly influenced by solvent polarity, being typically favored in nonpolar media.^21–23^

Among the many chiral anions employed for the resolution of metal complexes, TRISPHAT-based anions represent some of the most widely used and versatile resolving agents.^24^ However, other anionic frameworks have also proven to be effective. Borate-derived aromatic anions, such as 1,1′-binaphthalene-2,2′-diol borates have recently been employed as resolving agents in diverse ion-pair systems, including manganese complexes,^25^ Pt–Ag multinuclear clusters,^26^ asymmetric oxonium anions^27^ and barbaralane-based cages.^28^ Similarly, bis­(mandelato)­borate anions have been utilized in the separation of cobalt^29^ and rhodium^30^ chiral-at-metal complexes.

In this work, we demonstrate the chiral resolution of the atropisomeric cation **1** using a directed dynamic resolution strategy.^31–33^ This approach promotes the selective interconversion of enantiomeric cations under thermodynamic control, allowing the preferential enrichment of one enantiomer in solution. Upon crystallization, the enriched species affords diastereomerically pure crystals, effectively coupling dynamic solution-phase equilibration with selective solid-state resolution.

## Results and Discussion

In a recent study, we introduced an innovative atropisomeric silver-based complex exhibiting a linear geometry, with two *N*-heterocyclic carbene (NHC) ligands coordinating the metal center: [Ag­(NHC)_2_]^+^. Specifically, the title complex corresponds to [Ag­(Theo–CH_2_–Im–CH_2_–Theo)_2_]­[PF_6_] (Theo = theophylline, Im = imidazole), **1**[PF_6_], with silver coordinated at the C2 site of the NHC-imidazole ligand.^34^

In the solid state, cation **1** exhibits a C–Ag–C stereogenic axis owed to robust intramolecular π–π interactions between nucleobases. Therefore, distinct *R*_a_ and *S*_a_ enantiomers of **1** are identified (Figure 1). In solution, cation **1** retains its chiral information due to the stability provided by nucleobase–nucleobase interactions, thus existing as stable atropisomers, namely *R*_a_-**1** and *S*_a_-**1**. Additionally, NMR spectroscopy at low temperatures revealed decreased rotational dynamics of the *N*1-methyl group, further supporting conformational stability and restricted motion of the enantiomers. However, we reported that using this technique, the possibility of small-scale interconversion between the *R*_a_ and *S*_a_ enantiomers of **1**[PF_6_] could not be entirely ruled out.^34^

**Figure 1:**
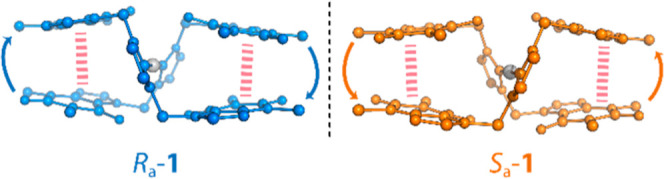
*R*_a_ and *S*_a_ enantiomers of cation **1**.

Initially, the chiral resolution of cation **1** was explored via diastereomeric salt formation with several enantiopure anions. In this work, both enantiomeric forms of the 1,1′-binaphthalene-2,2′-diol-borate anion, *R*_a_-BnB^–^ and *S*_a_-BnB^–^, were synthesized as sodium salts by adapting an established synthetic procedure.^35^ While the solid-state structure of Na­[*S*_a_-BnB]·5THF was recently reported by us,^34^ here we present the crystal structure of the *R*_a_-BnB^–^ enantiomer, namely, Na­[*R*_a_-BnB]·5THF (Figure 2). Upon addition of one of the enantiomers of Na­[BnB] into an acetonitrile solution of **1**[PF_6_], an immediate precipitation of **1**[BnB] occurred in high yield (>60%). This precipitate was then redissolved in dimethyl sulfoxide (DMSO), and upon slow diffusion of ethyl acetate (EtOAc) into the DMSO solution at room temperature over several days, formation of crystalline material was observed.

**Figure 2:**
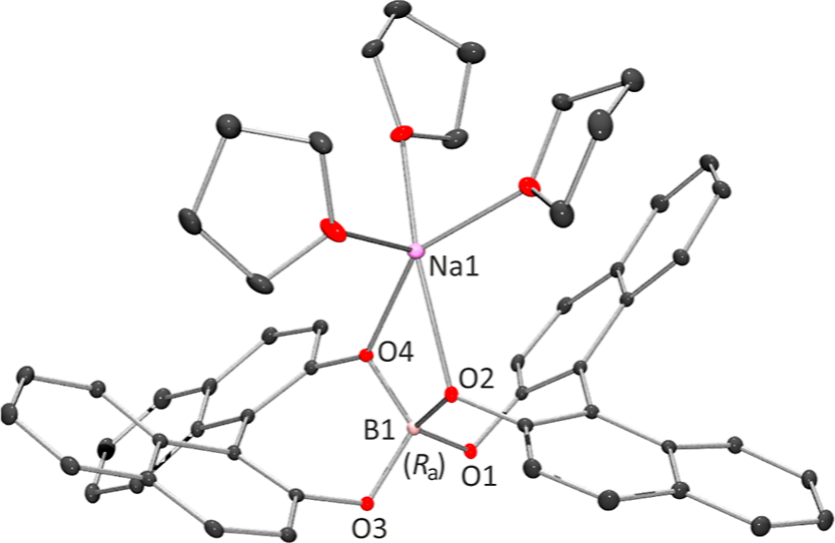
Coordination sphere of Na^+^ in Na­[*R*_a_-BnB]·5THF.

Despite repeated efforts, crystal structure analysis was hindered by poor-quality diffraction frames collected at the standard 42 mm detector distance, resulting in weak or undetectable diffraction peaks. Although a coherent unit cell was tentatively identified (orthorhombic *P*2_1_2_1_2_1_, *a* = 25.9 Å, *b* = 26.6 Å, *c* = 29.0 Å) the overall data remained insufficient for structure resolution.

Since the estimated unit cell parameters were not particularly large and, in principle, did not require modifications to the standard measurement setup, we explored the effect of increasing the crystal-to-detector distance as a potential strategy to enhance data quality. This exploratory approach was motivated by the well-defined prismatic morphology of the crystals and their exceptional optical quality under the microscope. Accordingly, selected crystals were mounted at varying distances from the diffractometer detector. This adjustment had a marked effect on diffraction quality. Increasing the detector distance improved spot separation and minimized diffraction overlap, although this usually implies reducing the intensity of weaker reflections. After evaluating multiple configurations, an intermediate distance of 80 mm was identified as the optimal compromise between resolution and signal intensity.

Under these optimized conditions, various **1**[BnB] crystals were successfully characterized by X-ray diffraction. First, a crystal containing the *S*_a_-BnB^–^ anion was selected and measured. As a result of the improved data quality, the structure and absolute configuration of both the *S*_a_-BnB^–^ anion and the associated *R*_a_-**1** cation (see below) was unambiguously determined. However, solvent molecules could not be resolved adequately. In order discard the solvent contribution to the structure factors, the SQUEEZE^36^ procedure implemented in PLATON^37^ was utilized. This analysis revealed a total of 2192 electrons within the unit cell. Considering that *Z* = 8, this corresponds to 274 electrons per cation **1**. Since sulfur atoms could be preliminary observed in the difference Fourier map, the number of solvent molecules was deduced to be one DMSO molecule and four EtOAc molecules per cation **1**, which matches perfectly the calculated electron count: (42 e^–^ DMSO) + (4 × 58 e^–^ EtOAc) = 274 e^–^. Thus, the molecular formula of the analyzed crystal was determined to be [*R*_a_-**1**]­[*S*_a_-BnB]·DMSO·4EtOAc.

Interestingly, preparations in which anion *S*_a_-BnB^–^ was incorporated led to the selective crystallization of cation *R*_a_-**1**, suggesting preferential cation–anion pairing of opposite chirality. The Flack parameter of 0.029(8) confirmed the absolute configuration of the [*R*_a_-**1**]­[*S*_a_-BnB]·DMSO·4EtOAc species. Conversely, when *R*_a_-BnB^–^ anion was utilized, crystals of the enantiomeric pair [*S*_a_-**1**]­[*R*_a_-BnB]·DMSO·4EtOAc were isolated, with a Flack parameter of 0.014(5), thereby validating the efficiency of the chiral resolution process in the solid state.

Molecular arrangements in [*R*_a_-**1**]­[*S*_a_-BnB]·DMSO·4EtOAc of cation *R*_a_-**1** and anion *S*_a_-BnB^–^ are similar to that reported by us for **1**[PF_6_] and Na­[BnB]·5THF, respectively.^34^ Intramolecular π–π interactions between theophylline scaffolds are around 3.8 Å, with angles ranging from 10.2° to 16.4°. A particularly noteworthy feature is the spatial organization of the cation–anion pairs within the crystal lattice. Two homochiral *R*_a_-**1** cations are stabilized by mutual 2-fold π–π interactions (Figure 3, center), stacked in an antiparallel arrangement. On the opposite sides, each *R*_a_-**1** cation interacts with two symmetry-related *S*_a_-BnB^–^ anions via strong π–π interactions (Figure 3, top and bottom). In both types of contacts, **1**···**1** and **1**···BnB, the π-ring separations are 3.4 Å, consistent with the strong and stabilizing nature of these interactions. The **1**···**1** contacts exhibit interplanar angles of 17.6° and 13.7°, indicating slightly greater distortion compared to the **1**···BnB interactions, where angles between naphthalene rings and nucleobases range from 1.6° to 8.3°. This arrangement leads to the formation of a hexagonal cage assembled by four *S*_a_-BnB^–^ anions, enclosing both *R*_a_-**1** cations in a well-defined host–guest system. Finally, [*S*_a_-**1**]­[*R*_a_-BnB]·DMSO·4EtOAc exhibits the expected analogous yet chiral packing motif, reflecting the mirror-image relationship between enantiomeric pairs.

**Figure 3:**
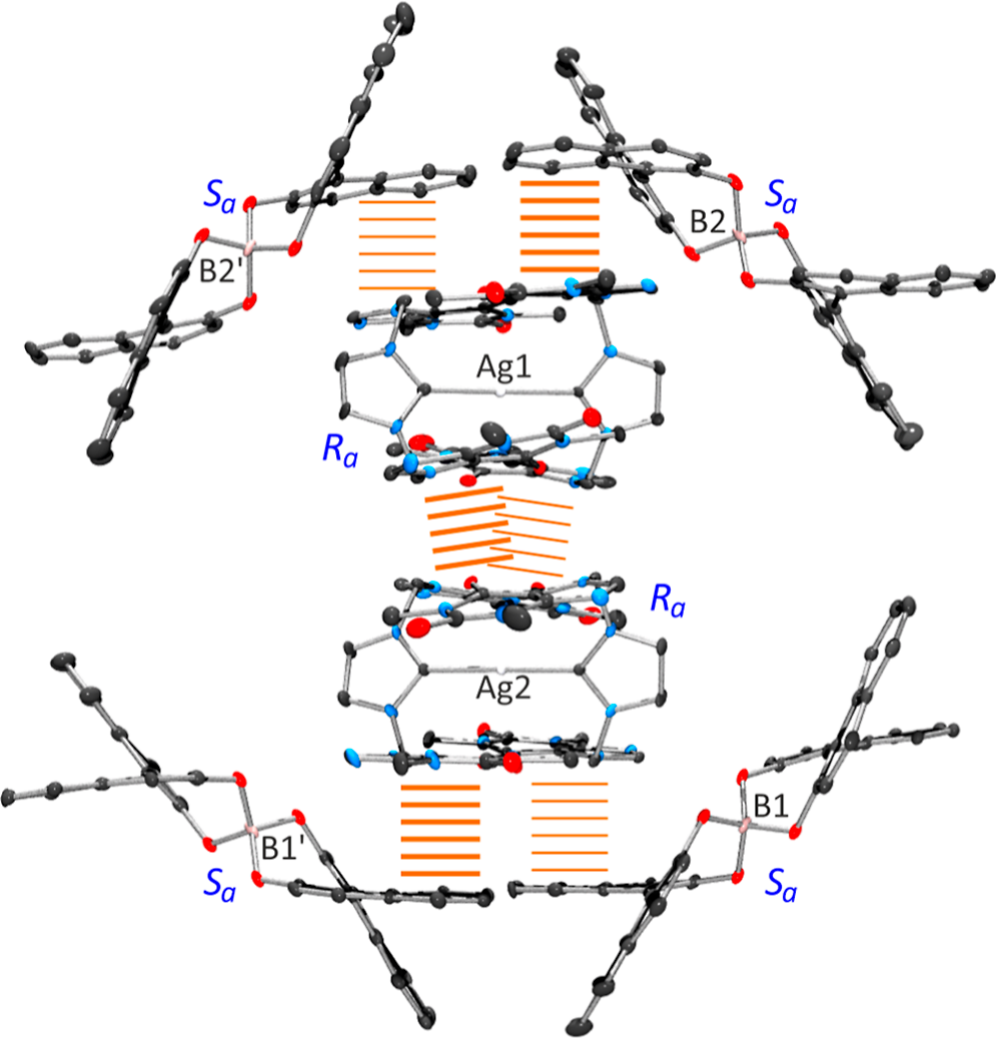
Anion–cation arrangement within the crystal lattice of [*R*_a_-**1**]­[*S*_a_-BnB]·DMSO·4EtOAc.

The unexpectedly high isolation yields of both isolated diastereomeric salts, [*S*_a_-**1**]­[*R*_a_-BnB] (66%) and [*R*_a_-**1**]­[*S*_a_-BnB] (61%), deviate from the typical outcomes observed in classical diastereomeric salt formation. To better understand this phenomenon, we investigated the solution behavior of cation **1** in the presence of counteranion BnB^–^ and compared it with that previously observed for **1**[PF_6_]. With this aim, we conducted a series of NMR experiments.

The ^1^H NMR spectrum of **1**[PF_6_] in CD_3_CN (Figure 4) exhibits a symmetric pattern on the NMR time scale.^34^ It displays characteristic signals for the nucleobase methyl groups (*N*1–CH_3_, 2.97 ppm; *N*3–CH_3_, 3.44) and H8 protons (8.12 ppm), the –CH_2_– bridging groups (6.66 ppm), and imidazole ring protons (7.65 ppm). Interestingly, analogous ^1^H NMR spectra in CD_3_CN of **1**[BnB] exhibit noteworthy differences (Figure 4, note that [*R*_a_-**1**]­[*S*_a_-BnB] and [*S*_a_-**1**]­[*R*_a_-BnB] yield identical ^1^H NMR spectra): The most striking one is the splitting of the –CH_2_– bridge signals (AB system) at room temperature, as the protons become diastereotopic (6.53 and 6.37 ppm). This arises from the rigidity of the surrounding environment of both –CH_2_– protons, which restricts rotation on the NMR time scale. This phenomenon is further confirmed by ^1^H–^13^C HSQC spectra (Supporting Information, Figure S15). Additionally, notable broadening of the *N*1–CH_3_ proton signal is observed even at room temperature (2.93 ppm), suggesting direct interactions between these methyl groups and nearby aromatic rings. It is worth noting that similar broadening was previously observed at a significantly lower temperature (233 K) for **1**[PF_6_] in CD_3_CN. Therefore, we hypothesize that the anionic chiral inductor BnB^–^ shifts the atropisomeric equilibrium *R*_a_-**1** ⇋ *S*_a_-**1** until cation **1** adopts the preferred conformation dictated by BnB^–^ and thereby inhibiting racemization in solution (see below).

**Figure 4:**
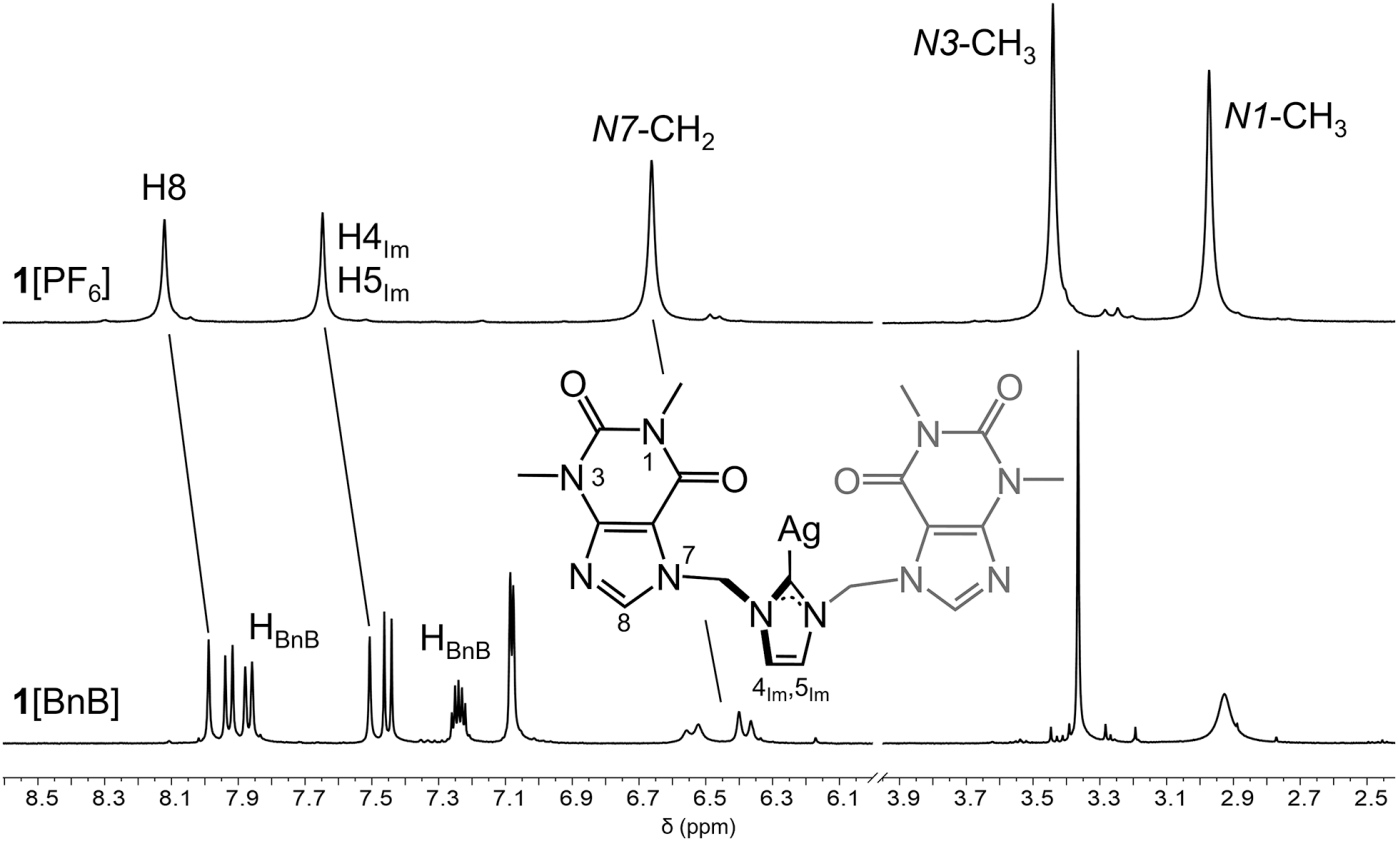
^1^H NMR spectra (CD_3_CN) of **1**[PF_6_] (298 K, top) and **1**[BnB] (298 K, bottom), and atomic numbering scheme including half cation **1**.

Variable temperature ^1^H NMR spectra (CD_3_CN) provide additional information regarding conformational stability of cation **1**. Notably, the *N*7–CH_2_ signals are sensitive to temperature changes (Figure 5). Upon cooling to 233 K, the AB system undergoes shielding consistent with a more rigid environment. Remarkably, one of the doublets is insensitive to temperature (the one located further lowfield), while the other is strongly affected, exhibiting significant shifts (Δδ = 0.26 ppm). We assume that the altered doublet is positioned closer to anion BnB^–^ and is therefore directly involved in π–π interactions, undergoing slight geometrical modifications during the stacking process. Furthermore, the *N*3–CH_3_ signal exhibits pronounced broadening at 233 K, further supporting its participation in π–π interactions. Importantly, no coalescence between both doublets of the AB system was detected even upon heating up to 343 K. These findings indicate that the atropisomeric conformation of cation **1** remains stable in solution even at elevated temperatures, reinforcing the robustness of the atropisomeric arrangement and suggesting that no racemization is expected to occur.

**Figure 5:**
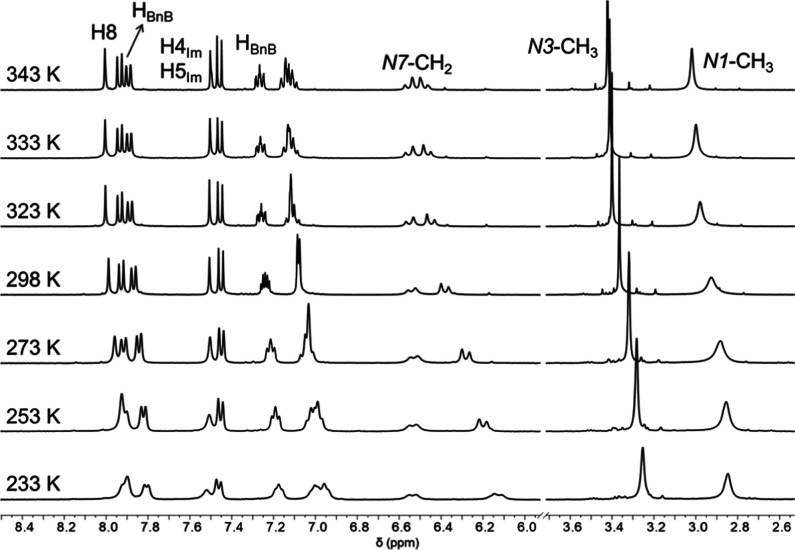
Variable temperature ^1^H NMR spectra of [*S*_a_-**1**]­[*R*_a_-BnB].

^1^H–^1^H NOESY spectrum recorded at room temperature confirms that the preferred atropisomeric conformation is retained in solution for both [*R*_a_-**1**]­[*S*_a_-BnB] and [*S*_a_-**1**]­[*R*_a_-BnB], as evidenced by cross peaks between H8 and H4_Im_, H5_Im_, H8 and *N*7–CH_2_, as well as between H4_Im_, H5_Im_ and *N*7–CH_2_ (Supporting Information, Figure S17). Upon cooling to 233 K in CD_3_CN, additional NOESY correlations emerge between the methyl groups and the aromatic rings of the borate anion, revealing interactions between both counterparts in solution (Supporting Information, Figure S27). These contacts are consistent with the π–π stacking motifs observed in the solid state structures.

^1^H DOSY NMR spectra were likewise recorded in CD_3_CN for both **1**[PF_6_] and **1**[BnB] (Supporting Information, Figures S31 and S32). The results suggest that cation **1** adopts a slightly more compact stacking arrangement in the presence of BnB^–^, likely due to the rigidity imposed by the −CH_2_– bridges, which appear as an AB system. In **1**[PF_6_], cation **1** displays a diffusion coefficient of 7.42 × 10^–10^ m^2^/s (r_H_ = 7.5 Å), whereas in **1**[BnB] the value increases to 7.99 × 10^–10^ m^2^/s (*r*_H_ = 7.0 Å), consistent with a more compact structure. For comparison, the imidazolium salt precursor shows a significantly higher diffusion coefficient of 1.59 × 10^–9^ m^2^/s (*r*_H_ = 3.5 Å), supporting the formation of larger, more rigid supramolecular assemblies in the metal complexes (Supporting Information, Figure S33).

Circular dichroism (CD) spectra recorded in CH_3_CN (Figure 6) and DMSO (Supporting Information, Figure S38) display mirror image profiles for [*R*_a_-**1**]­[*S*_a_-BnB] and [*S*_a_-**1**]­[*R*_a_-BnB], consistent with their enantiomeric relationship. Comparison with the spectra of the corresponding sodium salts, namely Na­[*R*_a_-BnB] and Na­[*S*_a_-BnB] (dotted lines in Figure 6), reveals slight differences in the 200–250 nm region, suggesting minor contributions from the chiral cations *R*_a_-**1** and *S*_a_-**1** (see UV–vis spectra, Supporting Information, Figures S36 and S37). However, the observed Cotton effects in the UV region are primarily attributed to the BnB^–^ anion,^25^ whose absorption dominates the CD spectra. Therefore, no bands could be assigned to cation **1**, as it does not exhibit absorption in the visible region. As a consequence, direct detection of the chiral information from cations *R*_a_-**1** and *S*_a_-**1** in solution via CD spectroscopy was not possible.

**Figure 6:**
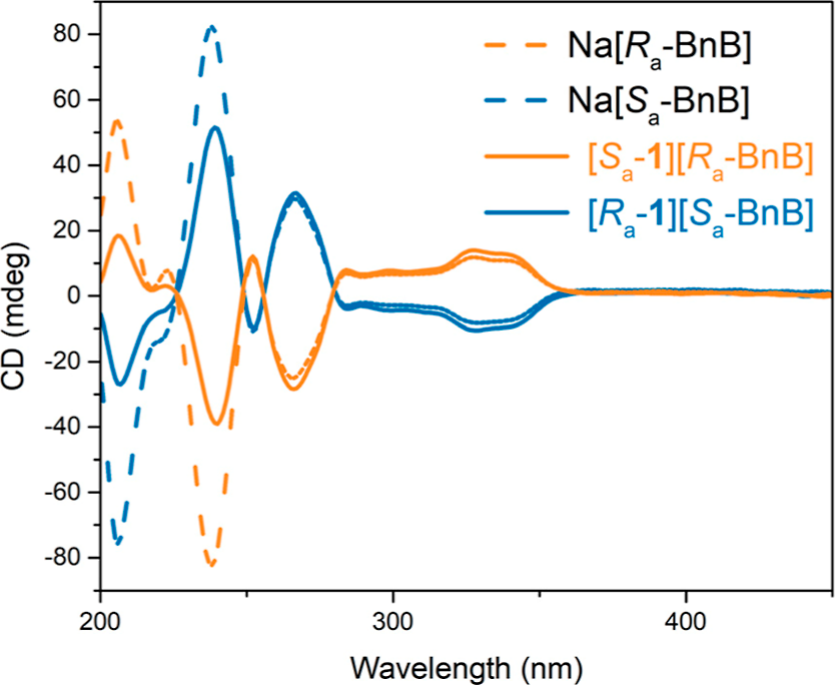
CD spectra of Na­[*R*_a_-BnB], Na­[*S*_a_-BnB], [*R*_a_-**1**]­[*S*_a_-BnB] and [*S*_a_-**1**]­[*R*_a_-BnB] (CH_3_CN, 10^–5^ M, 298 K).

The origin of the chiral resolution process is essentially driven by differences in noncovalent interactions, which govern the preferential stabilization of one enantiomer over the other. In this system, the enantiomeric *R*_a_-**1** ⇋ *S*_a_-**1** interconversion between atropisomers is modulated in terms of different π–π interaction affinities. Nucleobases are well-known for their propensity to stack via π–π interactions, involving their electron-deficient aromatic rings. The strength of such interactions typically increases when an electron-deficient ring interacts with an electron-rich counterpart, enhancing both attraction and stabilization.^38^ This principle is widely exploited in supramolecular chemistry, particularly in assemblies with controlled stereochemistry and in the design of molecular machines.^39,40^

In our system, we postulate that chiral resolution in solution is driven by selective π–π interactions between the electron-rich binaphthalene rings of the BnB^–^ borate anions and the electron-deficient aromatic rings of the nucleobases. This selective intermolecular interaction destabilizes the intramolecular π–π stacking between the two nucleobases of cation **1**, thereby lowering the energy barrier for enantiomeric *R*_a_-**1** ⇋ *S*_a_-**1** interconversion and facilitating the selective formation of a single atropisomer. The presence of BnB^–^ anions in solution facilitates this process by reducing the energetic cost associated with racemization, thus enabling a directed dynamic resolution (Figure 7). Notably, this interconversion occurs rapidly, as evidenced by NMR spectroscopy (Supporting Information, Figure S30). When a racemic mixture of cation **1** is treated with an enantiopure borate anion, a single enantiomer is selectively obtained in solution (eq 1).

1 $$\begin{array}{l} \left[R_{a}‐1⇋S_{a}‐1{]}^{+}+R_{a}‐{BnB}^{-}\to [S_{a}‐1][R_{a}‐BnB\right]\\ \left[R_{a}‐1⇋S_{a}‐1{]}^{+}+S_{a}‐{BnB}^{-}\to [R_{a}‐1][S_{a}‐BnB\right] \end{array}$$

**Figure 7:**
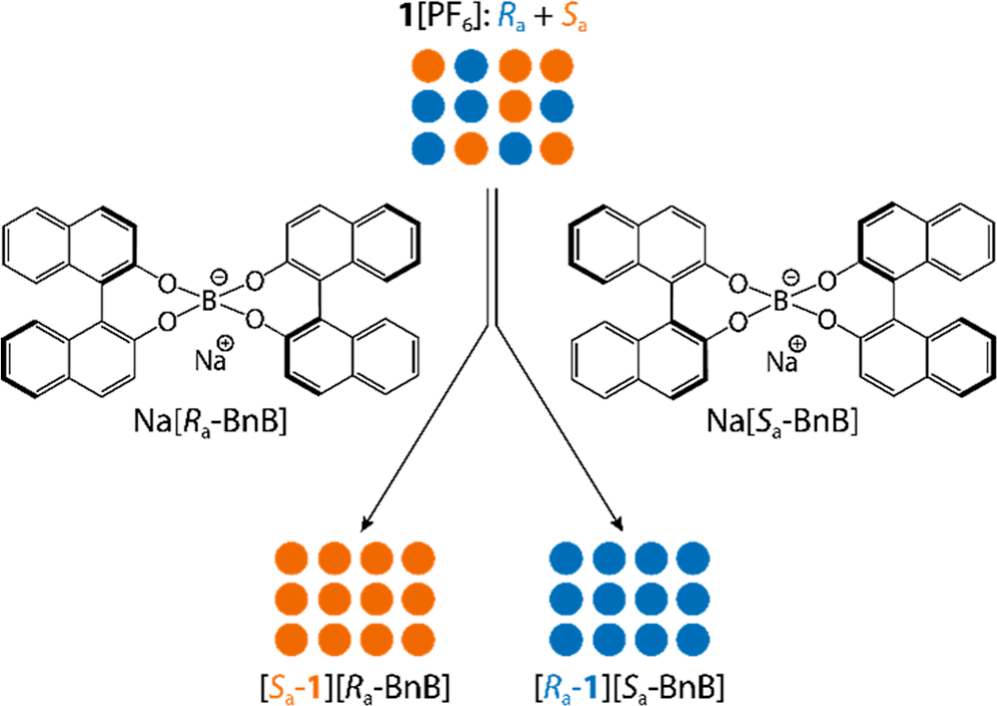
Conceptual depiction of the directed chiral resolution of cation **1** mediated by BnB^–^ anions.

Moreover, the enantiomeric species detected in solution corresponds to that isolated in the solid state, indicating that the chiral information observed in NOESY experiments is preserved throughout the resolution process.

## Conclusions

The enantiomeric resolution of the linear [Ag­(NHC)_2_]^+^ cation, *R*_a_-**1** and *S*_a_-**1**, has been successfully achieved through a directed dynamic resolution strategy, using enantiopure Na­[BnB] salts as chiral inducers. In solution, BnB^–^ anions selectively stabilize one atropisomer of cation **1** via selective π–π interactions between the electron-rich borate scaffold and the electron-deficient nucleobase rings: *R*_a_-BnB^–^ induces *S*_a_-**1**, while *S*_a_-BnB^–^ promotes *R*_a_-**1**. This stereoselective process was monitored by NMR and CD spectroscopies, confirming the exclusive formation of enantiopure cations. Furthermore, absolute configurations of the enantiomeric ion pairs were unambiguously determined by X-ray crystallographic analysis.

This chiral induction mechanism arises from directed stabilization of atropisomeric forms through electronically complementary aromatic interactions, which induce a shift in the *R*_a_-**1** ⇋ *S*_a_-**1** equilibrium at ambient temperature. In contrast to conventional diastereomeric salt formation, which separates stereoisomers via crystallization, our method enables reversible enantiomeric interconversion to occur in solution prior to crystallization through a dynamic thermodynamic resolution.

Chirality transfer from solution to the solid state highlights the crucial role of supramolecular interactions, particularly π–π stacking, in directing stereogenicity within configurationally labile systems The ability to induce the interconversion of otherwise stable enantiomers under mild conditions opens new opportunities for the design of dynamic systems in asymmetric recognition, resolution, and catalysis.

## Experimental Section

All reagents used in this study are from commercial origin and were and used without further purification. Glassware was dried at 120 °C before use. Unless otherwise specified, all reactions were conducted under aerobic conditions. Organic solvents were dried by standard procedures and distilled under argon prior to use, or obtained oxygen- and water-free from a Solvent Purification System (Innovative Technologies).

^1^H and ^13^C­{^1^H} NMR spectra were recorded using Bruker Avance 300 (300.13, 75.48, and 121.49 MHz, respectively) and Bruker Avance 400 (400.16, 100.61, and 161.98 MHz, respectively) spectrometers. Spectral assignments were achieved by combination of ^1^H–^1^H COSY, ^13^C­{^1^H}, ^13^C­{^1^H}-APT, and ^1^H–^13^C HSQC/HMBC experiments. NMR chemical shifts (expressed in parts per million) are referenced to residual solvent peaks (^1^H and ^13^C). Coupling constants, *J*, are given in hertz (Hz). UV–visible spectra in solution were recorded on a JASCO V-670 UV–vis spectrophotometer. CD spectra were recorded on a JASCO J-810 spectropolarimeter.

X-ray diffraction data of Na­[*R*_a_-BnB]·5THF (CCDC 2477508), [*R*_a_-**1**]­[*S*_a_-BnB]·DMSO·4EtOAc (CCDC 2477509), and [*S*_a_-**1**]­[*R*_a_-BnB]·DMSO·4EtOAc (2477510), were collected on a Bruker D8 Venture diffractometer, with graphite-monochromated Mo Kα radiation (λ = 0.71073 Å). Single crystals were mounted and coated with perfluoropolyether oil. Diffracted intensities were integrated with SAINT,^41^ and corrected of absorption effects was performed with a multiscan strategy by SADABS,^42,43^ both implemented in the APEX4 software package. Both structures were solved by direct methods with the software SHELXS^44^ and refined by full-matrix least-squares on *F* ^2^ with SHELXL program,^45^ and the WinGX system.^46^ To refine the crystal structure, the SQUEEZE^36^ procedure implemented in PLATON^37^ was used to remove the contribution of disordered solvent molecules from the electron density map (see main text for details).

Crystal data for compound Na­[*R*_a_-BnB]·5THF: C_60_H_64_BNaO_9_, *M*_r_ = 962.91, colorless prism, triclinic *P*1, *a* = 9.3238(4) Å, *b* = 11.0708(5) Å, *c* = 12.7064(6) Å, α = 86.3806(16)°, β = 79.8269(17)°, γ = 77.1408(16)°, *V* = 1258.19(10) Å^3^, *Z* = 1, *T* = 100(2) K, *D*_calcd_ = 1.271 cm^–3^, μ = 0.091 mm^–1^, absorption correction factors min. 0.952 max. 0.991, 84412 reflections, 12395 unique (*R*_int_ = 0.0356), 12067 observed, *R*_1_ = 0.0581 [*I* > 2σ­(*I*)], w*R*_2_(*F* ^2^) = 0.1634 (all data), GOF = 1.040. CCDC 2477508.

Crystal data for compound [*R*_a_-**1**]­[*S*_a_-BnB]·DMSO·4EtOAc: C_96_H_102_AgBN_20_O_21_S, *M*_r_ = 2022.71, colorless block, orthorhombic *P*2_1_2_1_2_1_, *a* = 25.9457(15) Å, *b* = 26.5537(16) Å, *c* = 29.0480(18) Å, *V* = 20013(2) Å^3^, *Z* = 8, *T* = 100(2) K, *D*_calcd_ = 1.343 cm^–3^, μ = 0.301 mm^–1^, absorption correction factors min. 0.893 max. 0.979, 401726 reflections, 49921 unique (*R*_int_ = 0.1251), 31858 observed, *R*_1_ = 0.0666 [*I* > 2σ­(*I*)], w*R*_2_(*F* ^2^) = 0.1904 (all data), GOF = 1.058. CCDC 2477509.

Crystal data for compound [*S*_a_-**1**]­[*R*_a_-BnB]·DMSO·4EtOAc: C_96_H_102_AgBN_20_O_21_S, *M*_r_ = 2022.71, colorless block, orthorhombic *P*2_1_2_1_2_1_, *a* = 25.8948(16)­Å, *b* = 26.6469(17) Å, *c* = 29.0326(18) Å, *V* = 20033(2) Å^3^, *Z* = 8, *T* = 100(2) K, *D*_calcd_ = 1.341 cm^–3^, μ = 0.301 mm^–1^, absorption correction factors min. 0.895 max. 0.977, 1144066 reflections, 49787 unique (*R*_int_ = 0.1531), 35832 observed, *R*_1_ = 0.0688 [*I* > 2σ­(*I*)], w*R*_2_(*F* ^2^) = 0.1897 (all data), GOF = 1.022. CCDC 2477510.

## Supplementary Material



## Notes

Synthetic procedures, NMR, HRMS, UV–vis, CD spectra, and X-ray data (PDF)
