# Supplementary material for: Directed Dynamic Resolution of an Atropisomeric Silver Complex in Solution: Role of π–π Interactions
Source: Inorg Chem. 2025 Nov 5;65(3):1773–9. doi: 10.1021/acs.inorgchem.5c03548 (PMC12848973; doi:10.1021/acs.inorgchem.5c03548)
Supplement: Supplementary file 1 [file ic5c03548_si_001.pdf]

SUPPORTING INFORMATION

Directed Dynamic Resolution of an Atropisomeric Silver Complex in Solution: Role of π–π Interactions

Alvaro Polo,*[a] Ricardo Rodríguez,[a] and Pablo J. Sanz Miguel*[a]

[a] Departamento de Química Inorgánica, Instituto de Síntesis Química y Catálisis Homogénea (ISQCH), Universidad de Zaragoza-CSIC. 50009 Zaragoza, Spain. Emails: alvaro.polo@unizar.es; pablo.sanz@unizar.es.

Contents

Synthesis and Characterization 2

NMR characterization of compounds [S_a-**1**][R_a-BnB] and [R_a-**1**][S_a-BnB] 3

HRMS spectra 19

UV-VIS spectra 20

CD spectra 21

X-Ray data 21

Synthesis and Characterization

All the reagents used in this work were purchased from commercial sources and used as received. Glassware was dried at 120 °C before use. Unless otherwise stated, all reactions were carried out under aerobic conditions. Crystallization procedures were performed using undried solvents. ^{1}H and $^{13}C\{^{1}H\}$ NMR spectra were recorded on Bruker Avance 300 (300.13 and 75.48 MHz, respectively) and Bruker Avance 400 (400.16 and 100.61 MHz, respectively) spectrometers. Spectral assignments were achieved by combination of ^{1}H-^{1}H COSY, $^{13}C\{^{1}H\}$-APT and ^{1}H-^{13}C HSQC/HMBC. NMR chemical shifts (expressed in parts per million) are referenced to residual solvent peaks (^{1}H and ^{13}C). Coupling constants, *J*, are given in hertz (Hz). High-resolution electrospray mass spectra (HRMS) were acquired using a MicroTOF-Q hybrid quadrupole time-of-flight spectrometer (Bruker Daltonics, Bremen, Germany). UV-visible spectra in solution were recorded on a JASCO V-670 UV-vis spectrophotometer. CD spectra were recorded on a Jasco J-810 spectropolarimeter.

Synthesis of [Ag(Theo-CH_2-Im-CH_2-Theo)$_2$][PF_6], (Theo = theophylline, Im = imidazole), 1[PF_6]

1[PF_6] was synthesized following a previously reported method.[SI-1]

Synthesis of sodium 1,1'-binaphthalene-2,2'-diol-borate, Na[BnB]

Both enantiomers of sodium 1,1'-binaphthalene-2,2'-diol-borate were synthesized using a modified procedure based on a previously reported method.[SI-2]

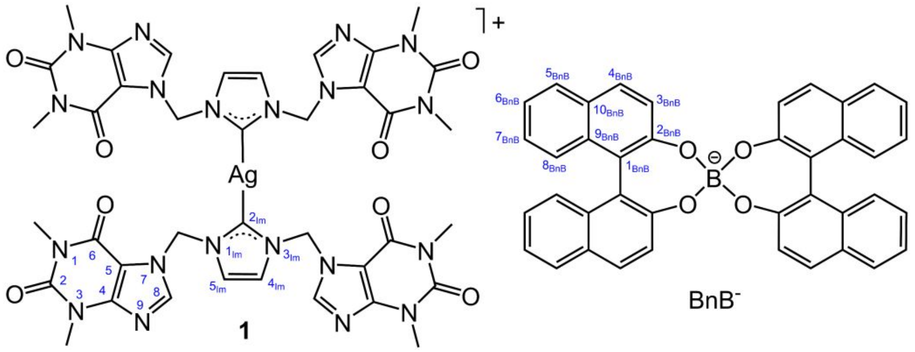

Synthesis of [Ag(Theo-CH_2-Im-CH_2-Theo)$_2$][1,1'-binaphthalene-2,2'-diol-borate], 1[BnB]

Synthesis of [S_a-1][R_a-BnB]: Na[R_a-BnB] (20 mg, 0.033 mmol) was added to a solution of **1**[PF_6] (19 mg, 0.016 mmol) in acetonitrile (1.5 mL), giving rise to a white precipitate. Upon stirring at room temperature for 45 minutes, the white solid was filtered, washed with diethyl ether (3 × 1 mL) and dried under vacuum to afford **1**[R_a-BnB] (17 mg, 0.011 mmol, 66 %). ^{1}H NMR (400 MHz, DMSO-d_6, 298 K): δ 8.47 (s, 4H, H8), 7.96 (d, $J_{H\text{-}H}$ = 8.7 Hz, 4H, $H4_{BnB}$), 7.92 (d, $J_{H\text{-}H}$ = 8.1 Hz, 4H, $H5_{BnB}$), 7.88 (br, 4H, $H4_{Im}$ + $H5_{Im}$), 7.33 (d, $J_{H\text{-}H}$ = 8.7 Hz, 4H, $H3_{BnB}$), 7.27 (m, 4H, $H6_{BnB}$), 7.13 (m, 8H, $H7_{BnB}$ + $H8_{BnB}$), 6.74 (br, 8H, *N7*-CH_2), 3.38 (s, 12H, *N3*-CH_3), 2.80 (s, 12H, *N1*-CH_3). $^{13}C\{^{1}H\}$ NMR (100 MHz, DMSO-d_6, 298 K): δ 156. 1 ($C2_{BnB}$), 154.5 (C6), 150.6 (C2), 148.7 (C4), 143.2 (C8), 132.8 ($C10_{BnB}$), 128.9 ($C9_{BnB}$), 128.2 ($C4_{BnB}$), 128.0 ($C5_{BnB}$), 125.8 ($C8_{BnB}$), 124.8 ($C7_{BnB}$), 124.5

($C3_{BnB}$), 122.4 ($C4_{Im}$ + $C5_{Im}$ + $C6_{BnB}$), 121.8 ($C1_{BnB}$), 105.0 (C5), 58.8 (*N7*-C), 29.6 (*N3*-C), 27.2 (*N1*-C). ^{1}H NMR (400 MHz, CD_3CN, 298 K): δ 7.98 (s, 4H, H8), 7.92 (d, $J_{H\text{-}H}$ = 8.7 Hz, 4H, $H4_{BnB}$), 7.86 (d, $J_{H\text{-}H}$ = 8.1 Hz, 4H, $H5_{BnB}$), 7.50 (s, 4H, $H4_{Im}$ + $H5_{Im}$), 7.45 (d, $J_{H\text{-}H}$ = 8.7 Hz, 4H, $H3_{BnB}$), 7.23 (m, 4H, $H6_{BnB}$), 7.07 (m, 8H, $H7_{BnB}$ + $H8_{BnB}$), 6.53 (d, $J_{H\text{-}H}$ = 14.1 Hz, 4H, *N7*-CH_2), 6.37 (d, $J_{H\text{-}H}$ = 14.1 Hz, 4H, *N7*-CH_2), 3.36 (s, 12H, *N3*-CH_3), 2.93 (br, 12H, *N1*-CH_3). ^{13}C{^{1}H} NMR (100 MHz, CD_3CN, 298 K): δ 157.0 ($C2_{BnB}$), 156.0 (C6), 152.0 (C2), 150.2 (C4), 143.4 (C8), 134.0 ($C10_{BnB}$), 130.4 ($C9_{BnB}$), 129.5 ($C4_{BnB}$), 129.0 ($C5_{BnB}$), 126.9 ($C8_{BnB}$), 125.8 ($C7_{BnB}$), 125.6 ($C3_{BnB}$), 123.5 ($C6_{BnB}$), 123.2 ($C4_{Im}$ + $C5_{Im}$), 123.1 ($C1_{BnB}$), 106.4 (C5), 60.1 (*N7*-C), 30.3 (*N3*-C), 28.0 (*N1*-C).

Synthesis of [*R*$_a$-1][*S*$_a$-BnB]: Na[S_a-BnB] (20 mg, 0.033 mmol) was added to a solution of **1**[PF_6] (19 mg, 0.016 mmol) in acetonitrile (1.5 mL), giving rise to a white precipitate. Upon stirring at room temperature for 45 minutes, the white solid was filtered, washed with diethyl ether (3 × 1 mL) and dried under vacuum to afford **1**[S_a-BnB] (16 mg, 0.010 mmol, 61 %). ^{1}H NMR (400 MHz, DMSO-d_6, 298 K): δ 8.48 (s, 4H, H8), 7.96 (d, $J_{H\text{-}H}$ = 8.6 Hz, 4H, $H4_{BnB}$), 7.92 (d, $J_{H\text{-}H}$ = 8.2 Hz, 4H, $H5_{BnB}$), 7.89 (br, 4H, $H4_{Im}$ + $H5_{Im}$), 7.33 (d, $J_{H\text{-}H}$ = 8.6 Hz, 4H, $H3_{BnB}$), 7.26 (m, 4H, $H6_{BnB}$), 7.13 (m, 8H, $H7_{BnB}$ + $H8_{BnB}$), 6.75 (br, 8H, *N7*-CH_2), 3.39 (s, 12H, *N3*-CH_3), 2.81 (s, 12H, *N1*-CH_3). ^{1}H NMR (400 MHz, CD_3CN, 298 K): δ 7.99 (s, 4H, H8), 7.94 (d, $J_{H\text{-}H}$ = 8.7 Hz, 4H, $H4_{BnB}$), 7.89 (d, $J_{H\text{-}H}$ = 8.2 Hz, 4H, $H5_{BnB}$), 7.51 (s, 4H, $H4_{Im}$ + $H5_{Im}$), 7.46 (d, $J_{H\text{-}H}$ = 8.7 Hz, 4H, $H3_{BnB}$), 7.26 (m, 4H, $H6_{BnB}$), 7.10 (m, 8H, $H7_{BnB}$ + $H8_{BnB}$), 6.54 (d, $J_{H\text{-}H}$ = 14.2 Hz, 4H, *N7*-CH_2), 6.39 (d, $J_{H\text{-}H}$ = 14.2 Hz, 4H, *N7*-CH_2), 3.37 (s, 12H, *N3*-CH_3), 2.94 (br, 12H, *N1*-CH_3).

HRMS (ESI+, DMSO-d_6/CH_3CN, m/z): calculated for $C_{38}H_{40}N_{20}O_8Ag$, 1011.2383 [**1**]$^+$; experimental, 1011.2413 [**1**]$^+$. HRMS (ESI–, DMSO-d_6/CH_3CN, m/z): calculated for $C_{40}H_{24}BO_4$, 579.1780 [BnB]$^-$; experimental, 579.1748 [BnB]$^-$.

NMR characterization of compounds [*S*$_a$-1][*R*$_a$-BnB] and [*R*$_a$-1][*S*$_a$-BnB]

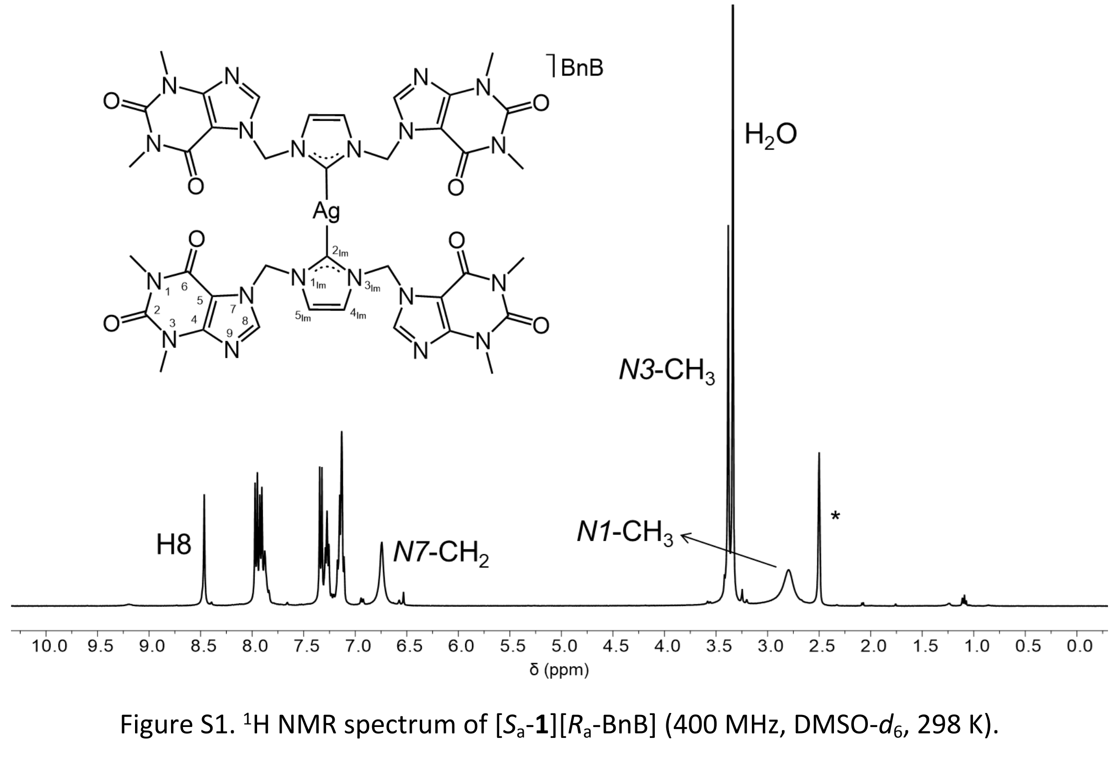

Figure S1. ^{1}H NMR spectrum of [S_a-**1**][R_a-BnB] (400 MHz, DMSO-d_6, 298 K).

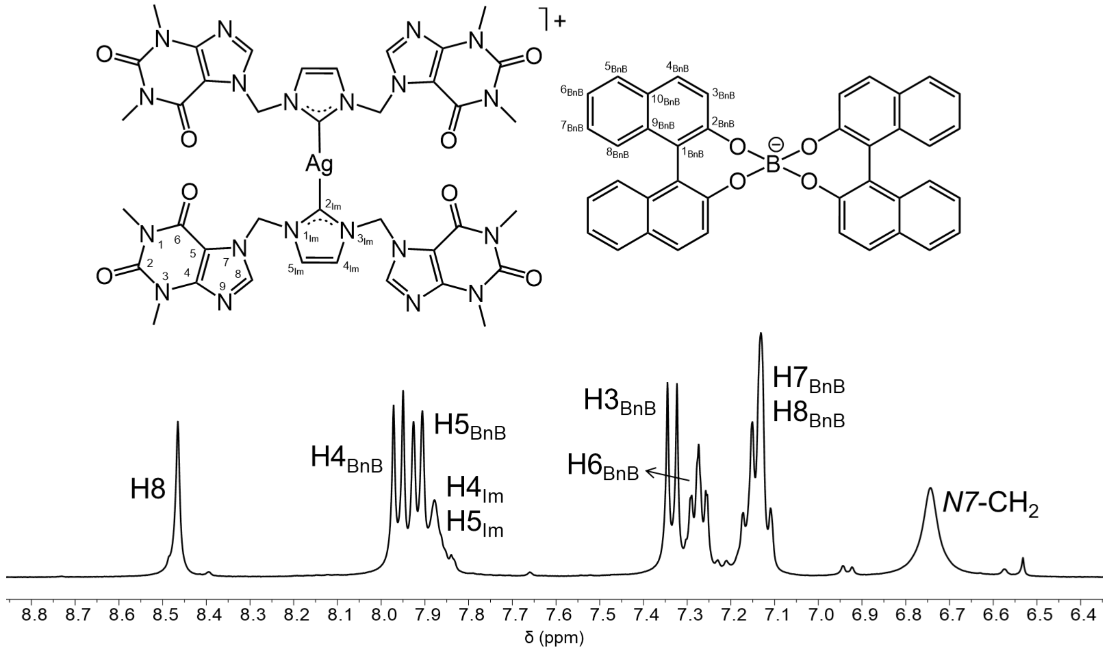

Figure S2. Aromatic region of the ^{1}H NMR spectrum of [S_a-**1**][R_a-BnB] (400 MHz, DMSO-d_6, 298 K).

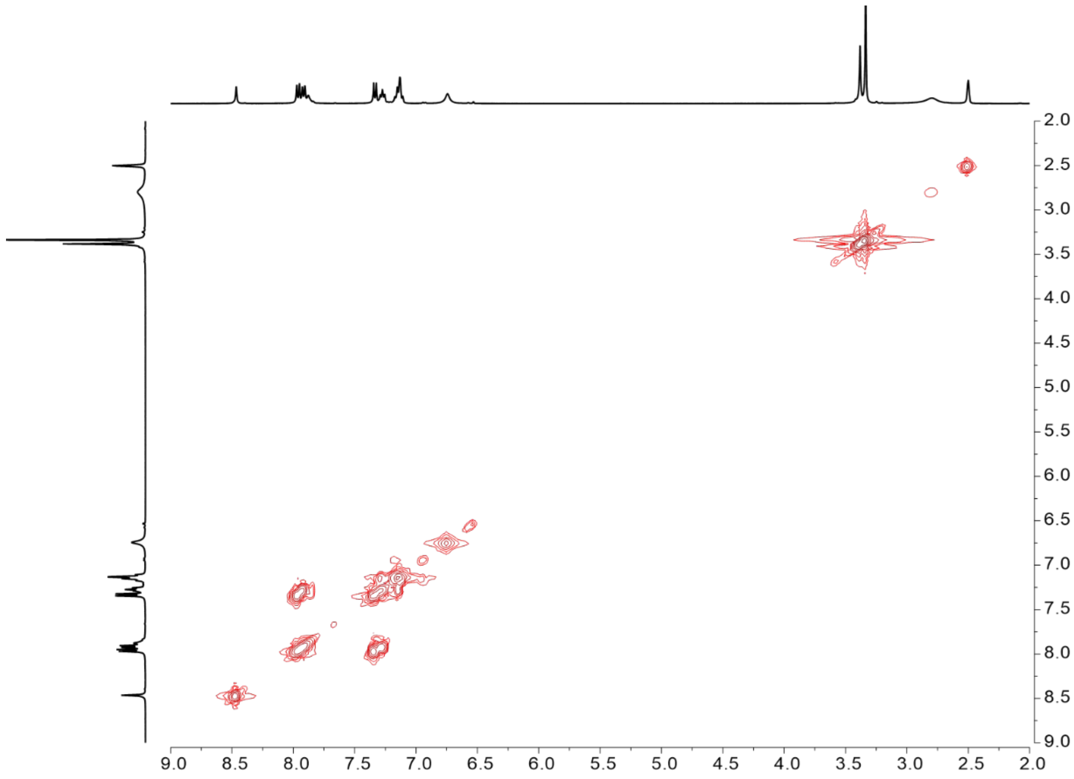

Figure S3. ^{1}H-^{1}H COSY NMR spectrum of [S_a-**1**][R_a-BnB] (DMSO-d_6, 298 K).

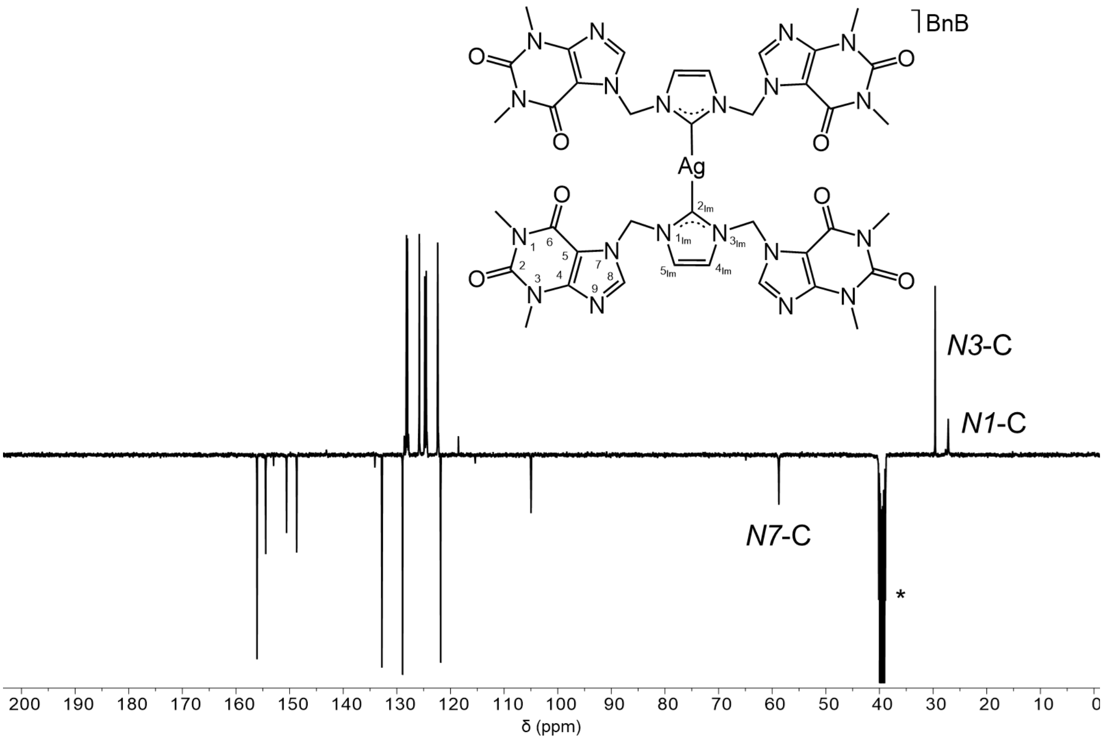

Figure S4. $^{13}C\{^{1}H\}$-APT NMR spectrum of [S_a-**1**][R_a-BnB] (100 MHz, DMSO-d_6, 298 K).

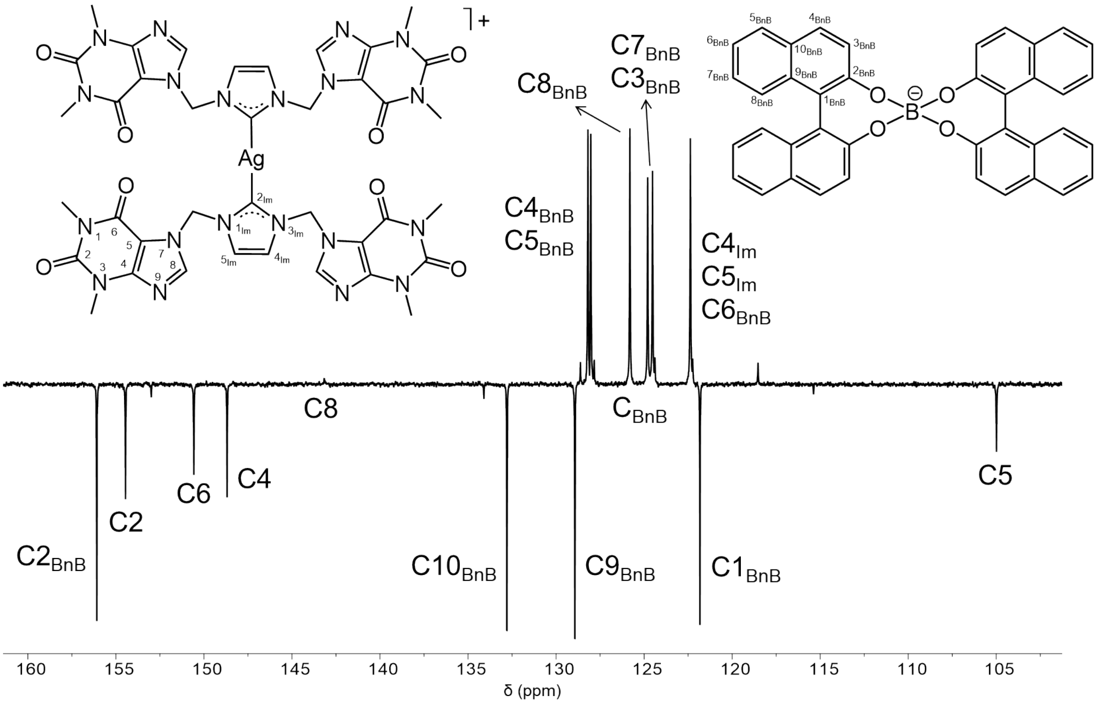

Figure S5. Aromatic region of the $^{13}C\{^{1}H\}$-APT NMR spectrum of [S_a-**1**][R_a-BnB] (100 MHz, DMSO-d_6, 298 K).

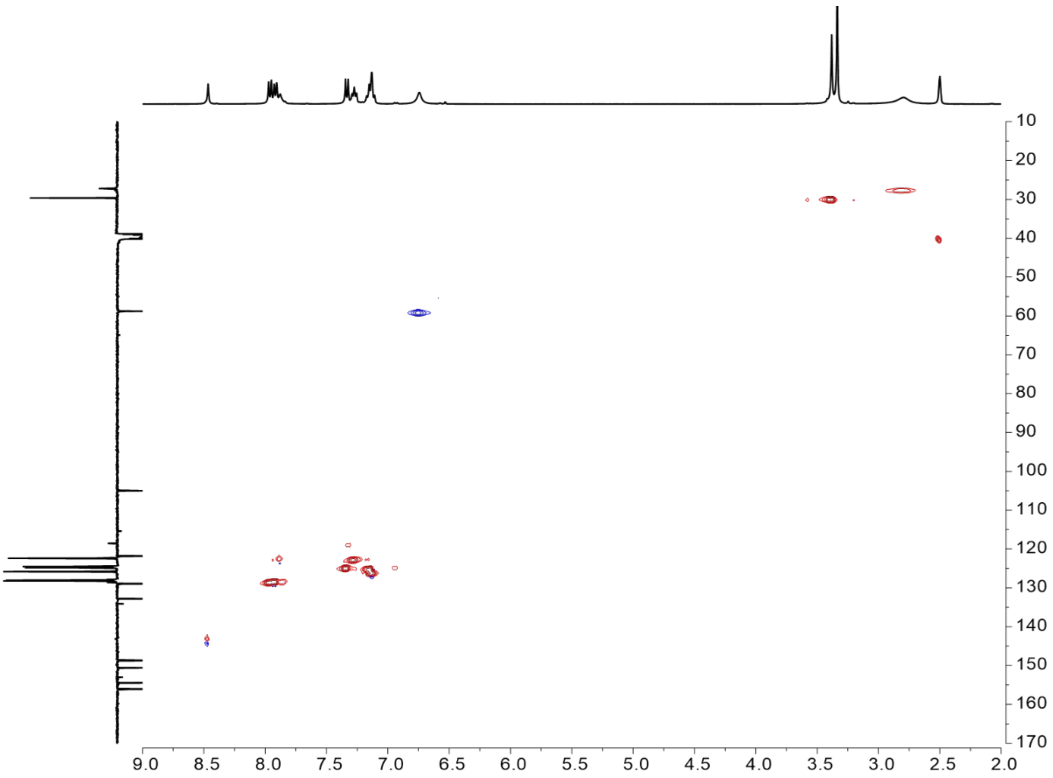

Figure S6. ^{1}H-^{13}C HSQC NMR spectrum of [S_a-**1**][R_a-BnB] (DMSO-d_6, 298 K).

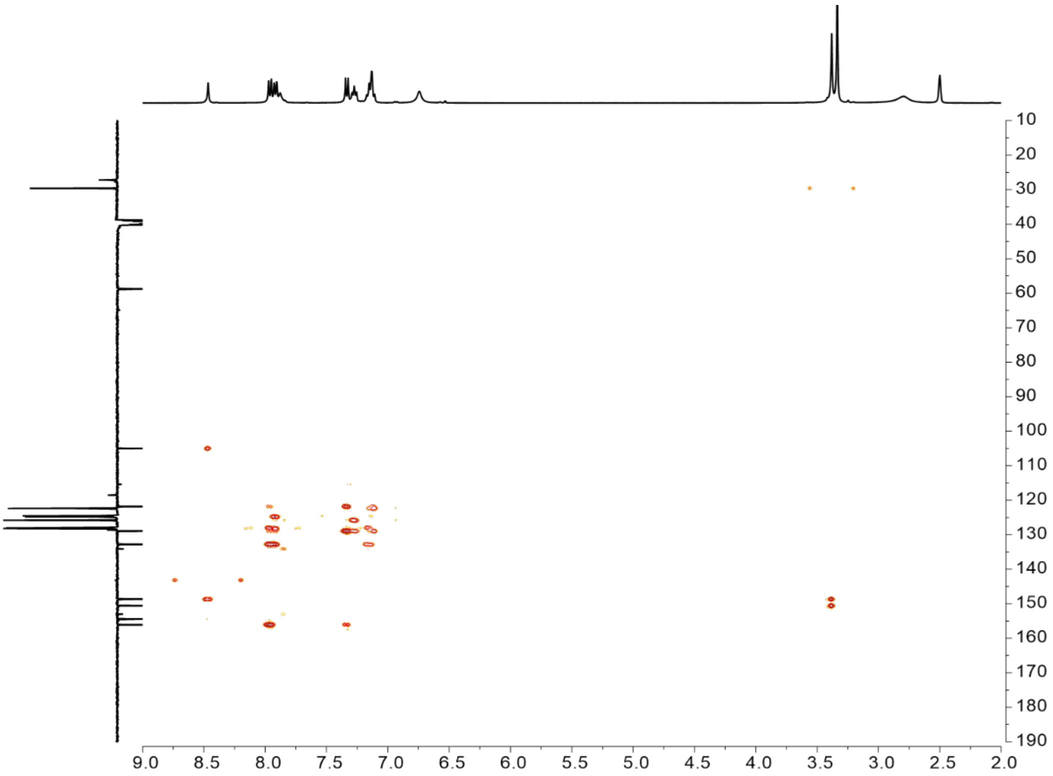

Figure S7. ^{1}H-^{13}C HMBC NMR spectrum of [S_a-**1**][R_a-BnB] (DMSO-d_6, 298 K).

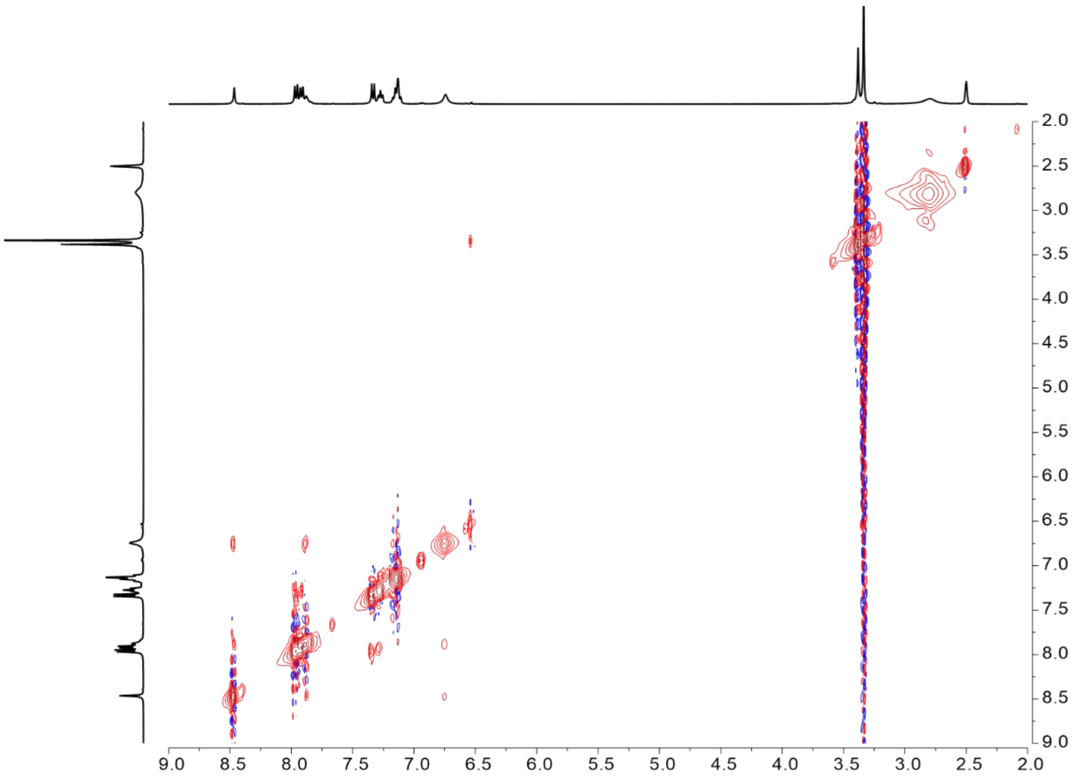

Figure S8. ^{1}H-^{1}H NOESY NMR spectrum of [S_a-**1**][R_a-BnB] (DMSO-d_6, 298 K).

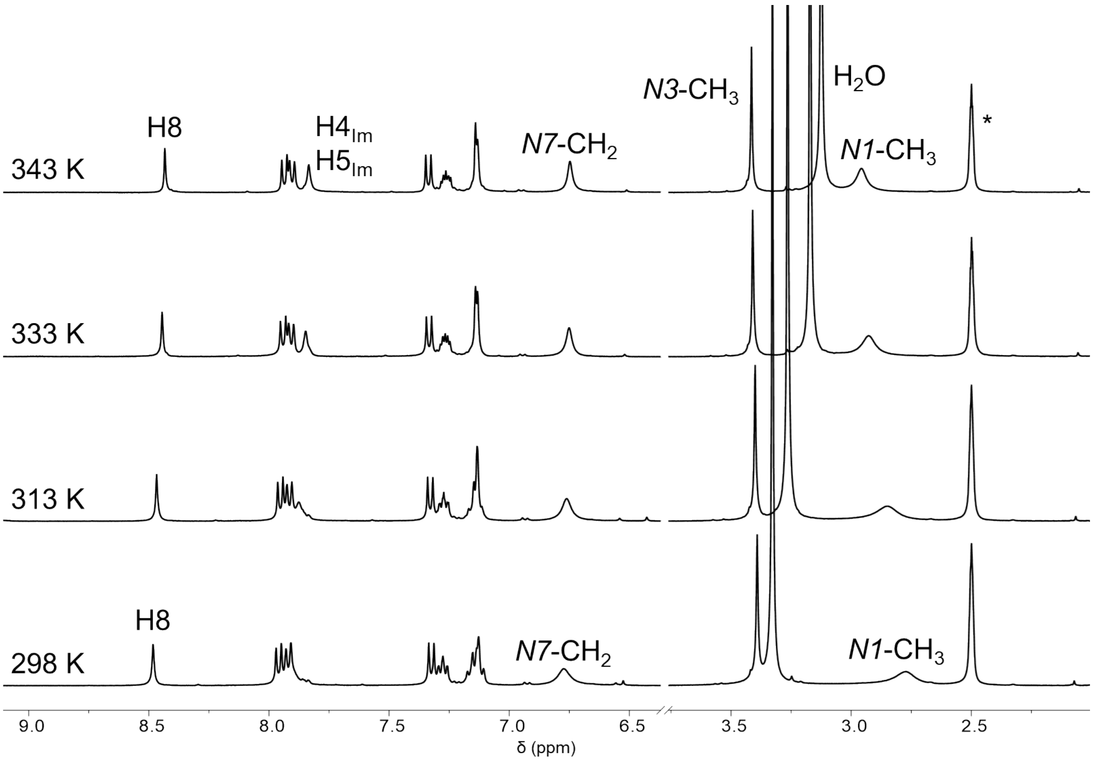

Figure S9. Variable temperature ^{1}H NMR spectra of [S_a-**1**][R_a-BnB] (100 MHz, DMSO-d_6).

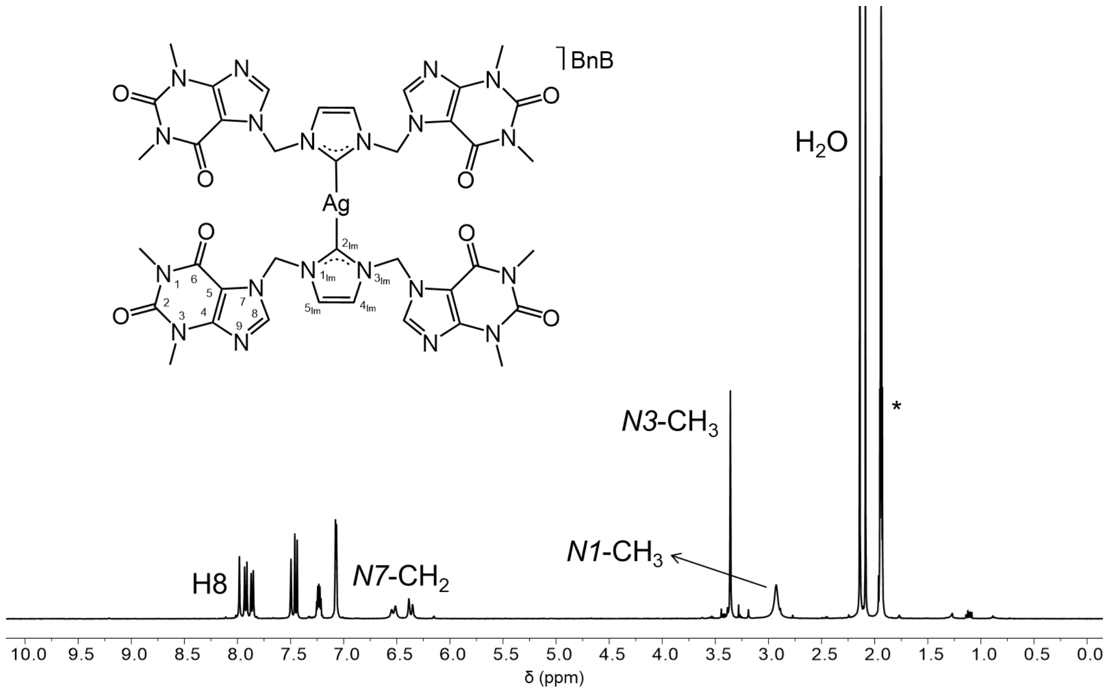

Figure S10. ^{1}H NMR spectrum of [S_a-**1**][R_a-BnB] (100 MHz, CD_3CN, 298 K).

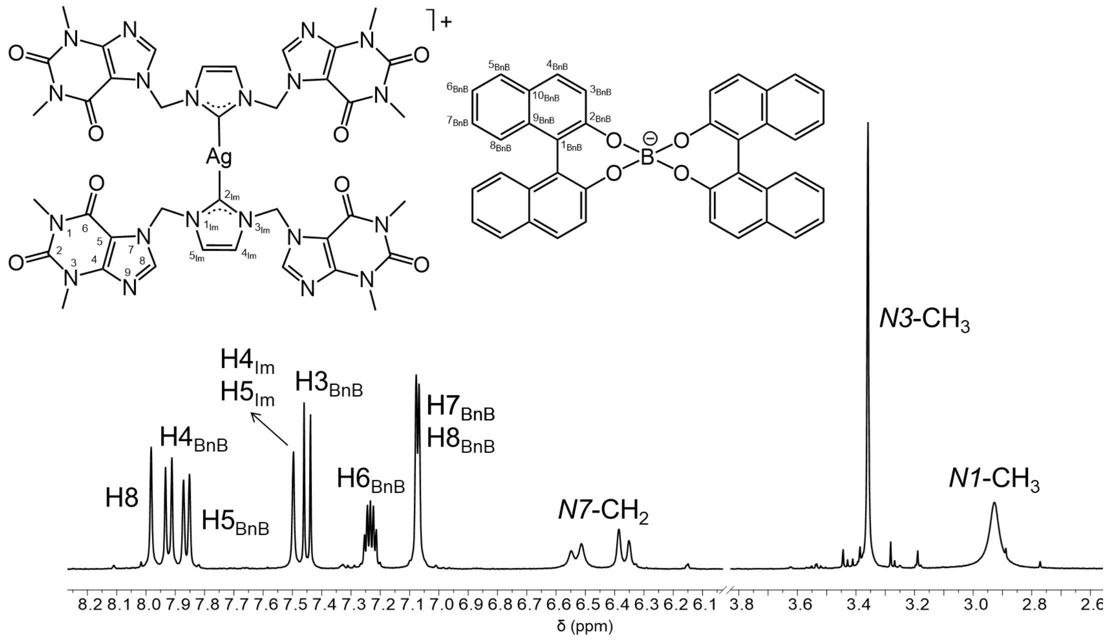

Figure S11. Aromatic region of the ^{1}H NMR spectrum of [S_a-**1**][R_a-BnB] (100 MHz, CD_3CN, 298 K).

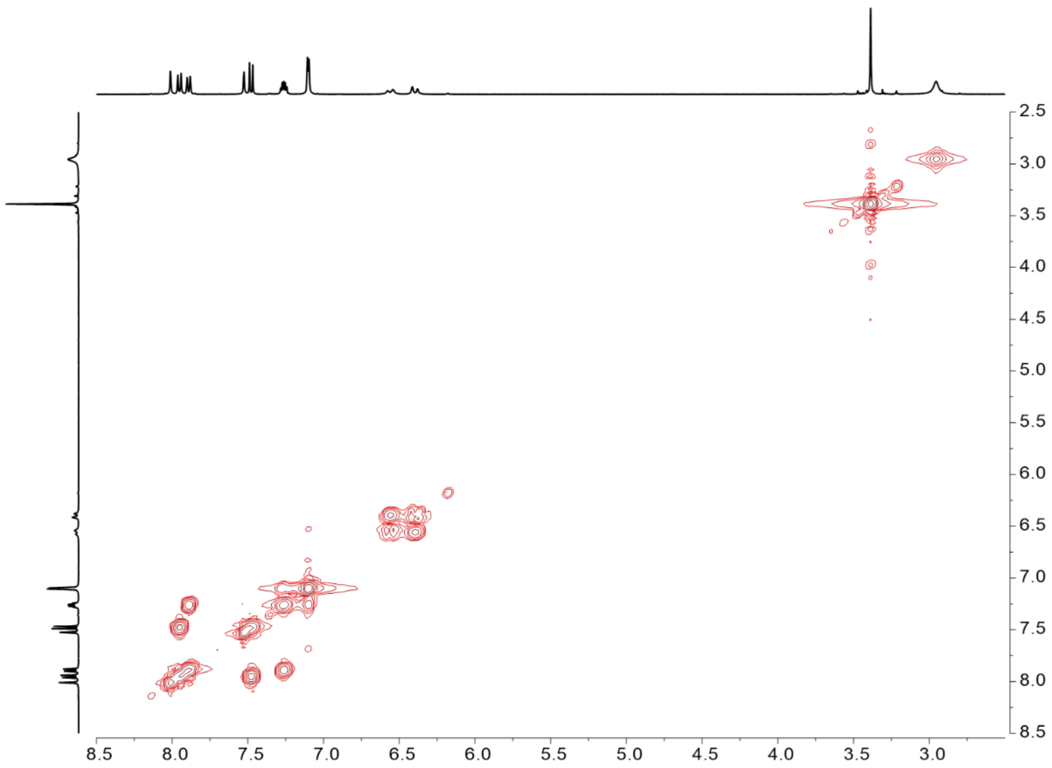

Figure S12. ^{1}H-^{1}H COSY NMR spectrum of [S_a-**1**][R_a-BnB] (CD_3CN, 298 K).

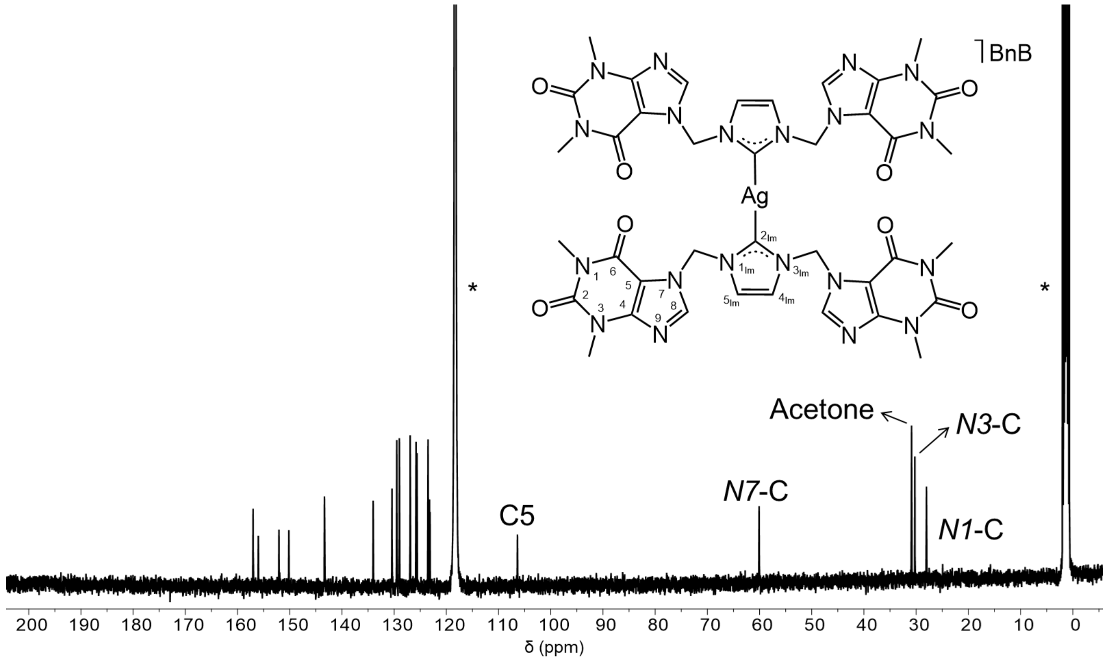

Figure S13. $^{13}C\{^{1}H\}$-APT NMR spectrum of [S_a-**1**][R_a-BnB] (100 MHz, CD_3CN, 298 K).

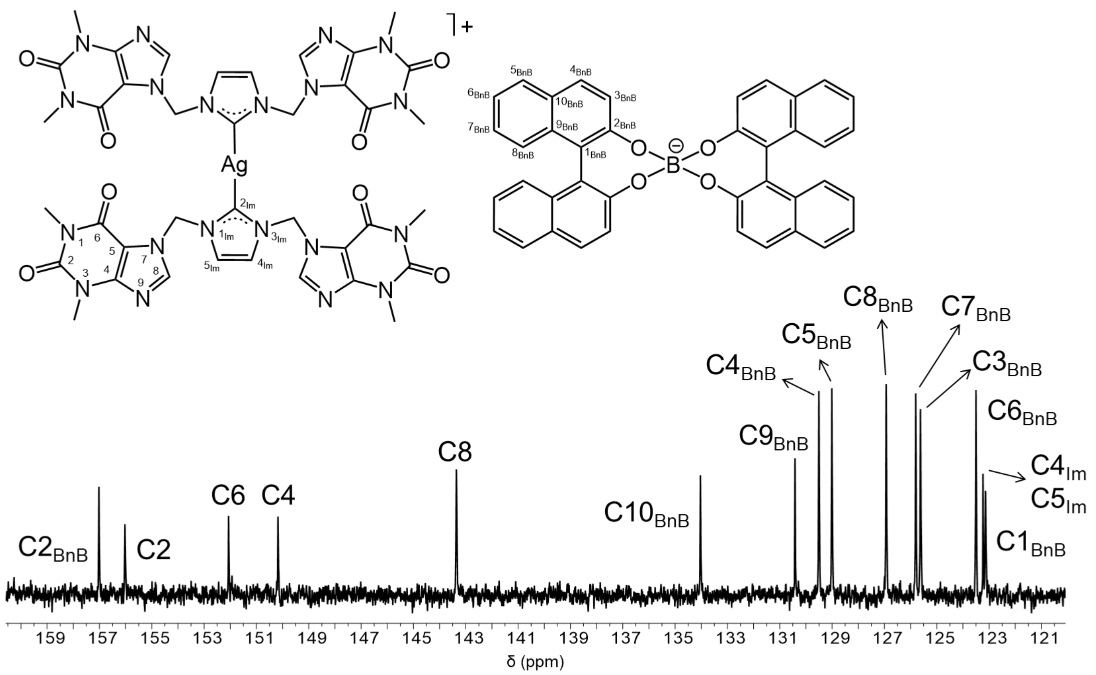

Figure S14. Aromatic region of the $^{13}C\{^{1}H\}$-APT NMR spectrum of [S_a-**1**][R_a-BnB] (100 MHz, CD_3CN, 298 K).

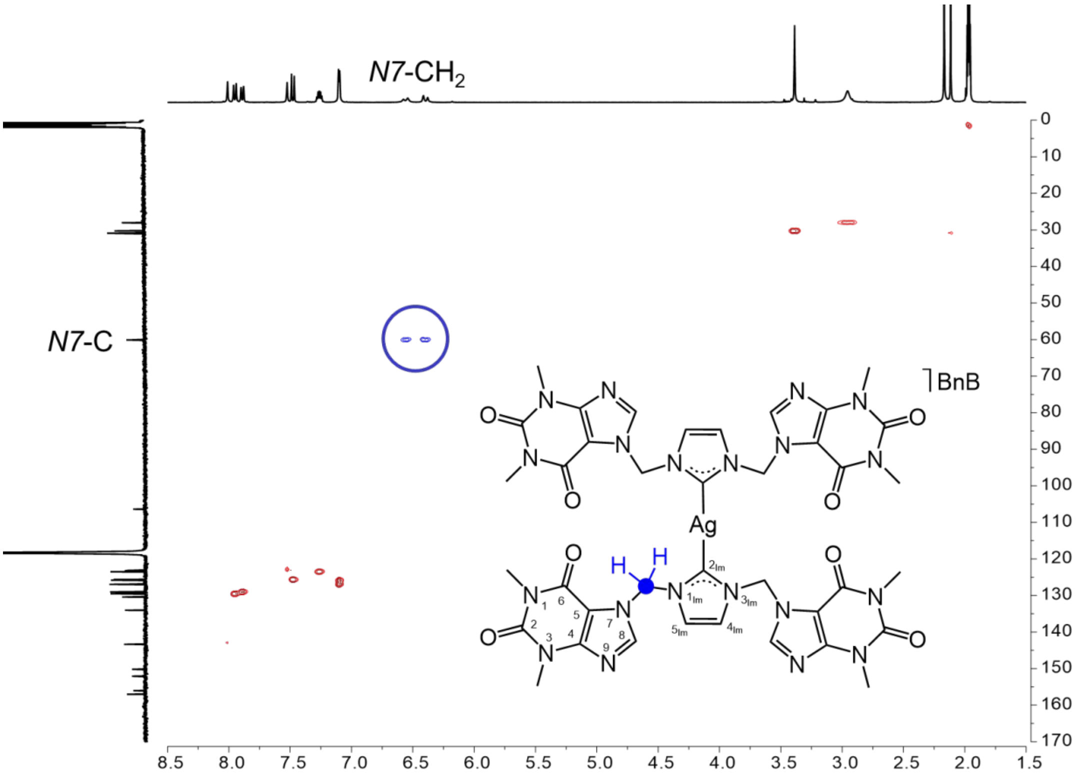

Figure S15. ^{1}H-^{13}C HSQC NMR spectrum of [S_a-**1**][R_a-BnB] (CD_3CN, 298 K).

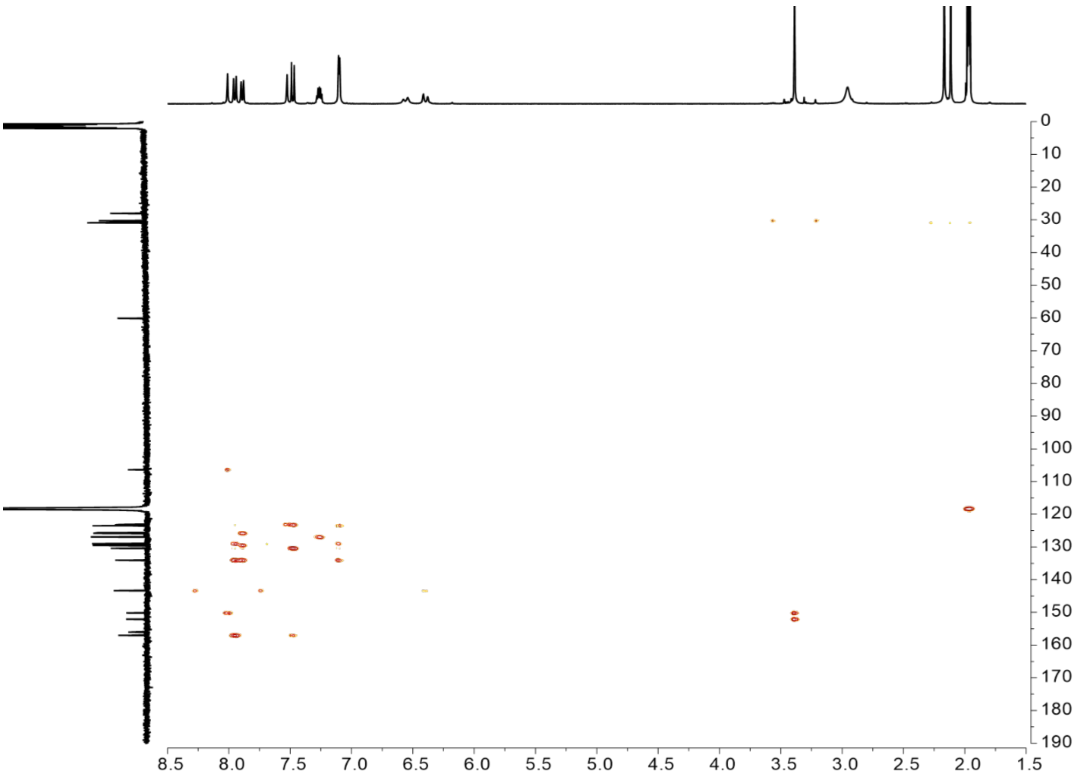

Figure S16. ^{1}H-^{13}C HMBC NMR spectrum of [S_a-**1**][R_a-BnB] (CD_3CN, 298 K).

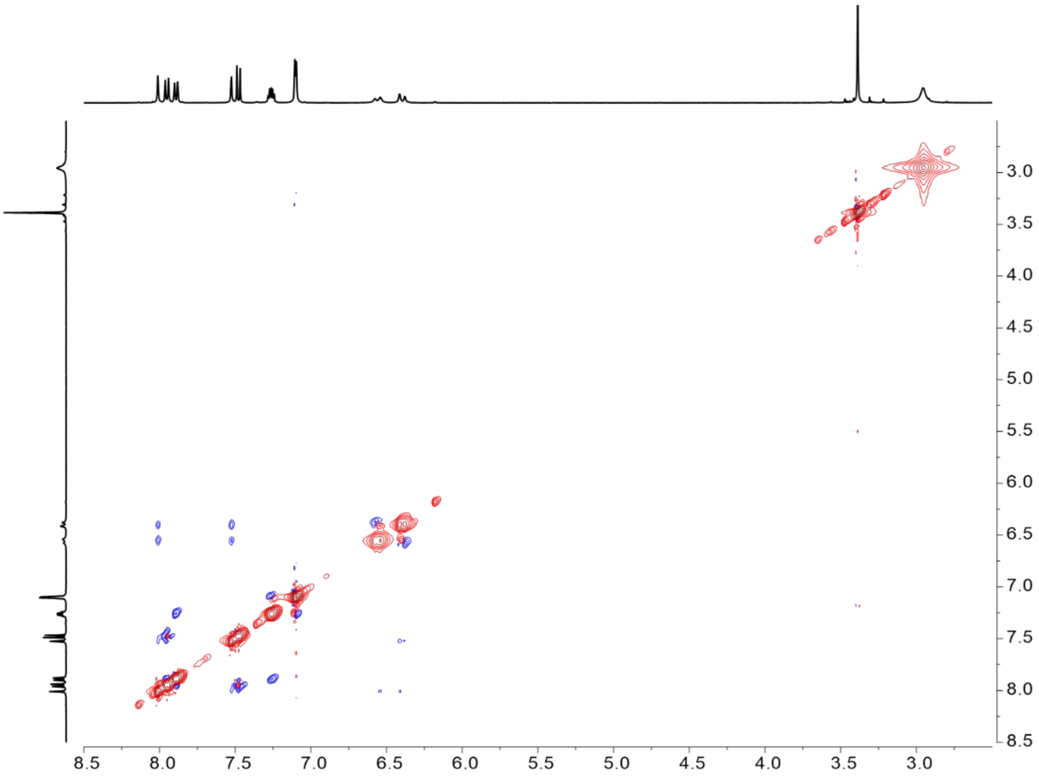

Figure S17. ^{1}H-^{1}H NOESY NMR spectrum of [S_a-**1**][R_a-BnB] (CD_3CN, 298 K).

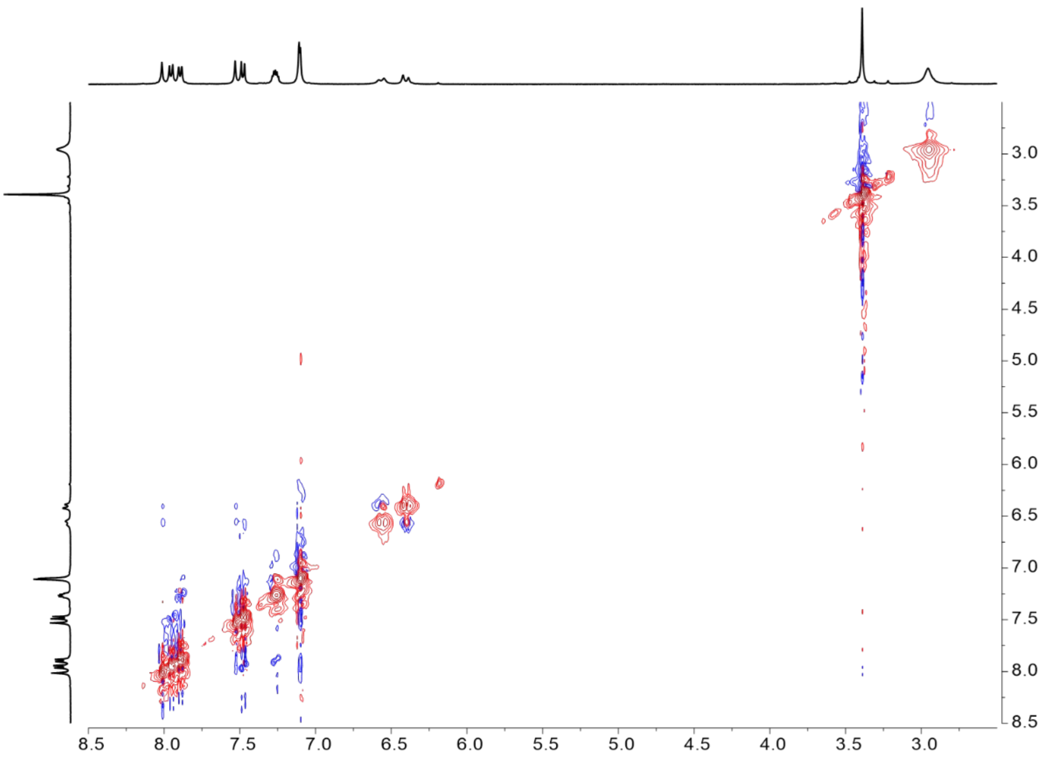

Figure S18. ^1H-^1H ROESY NMR spectrum of [S_a-**1**][R_a-BnB] (CD_3CN, 298 K).

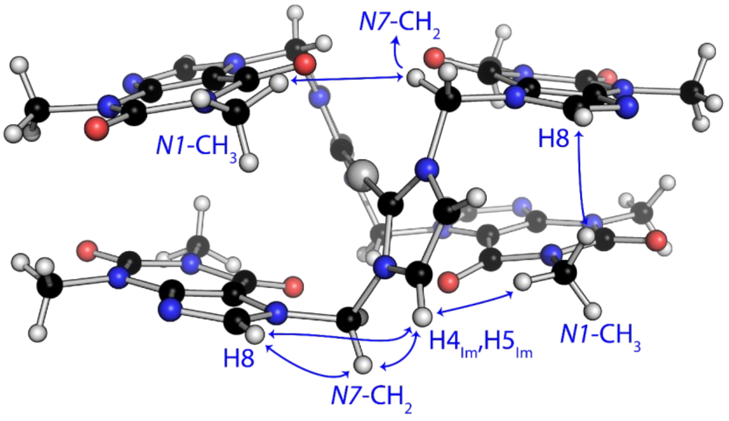

Figure S19. Interplay between protons in the ^1H-^1H NOESY NMR spectrum of [S_a-**1**][R_a-BnB].

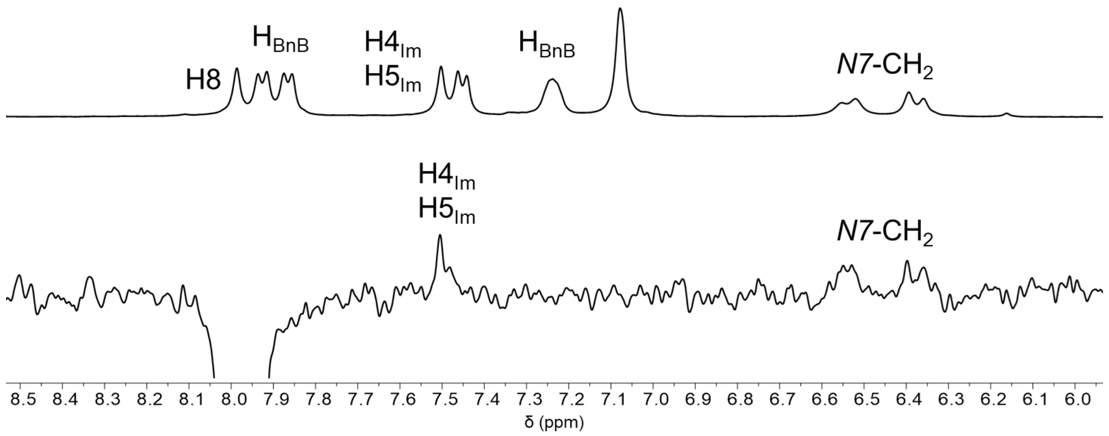

Figure S20. Selected region in the [1]H 1D-selNOE of [S_a-**1**][R_a-BnB] upon irradiation at 8.01 ppm (CD_3CN, 298 K).

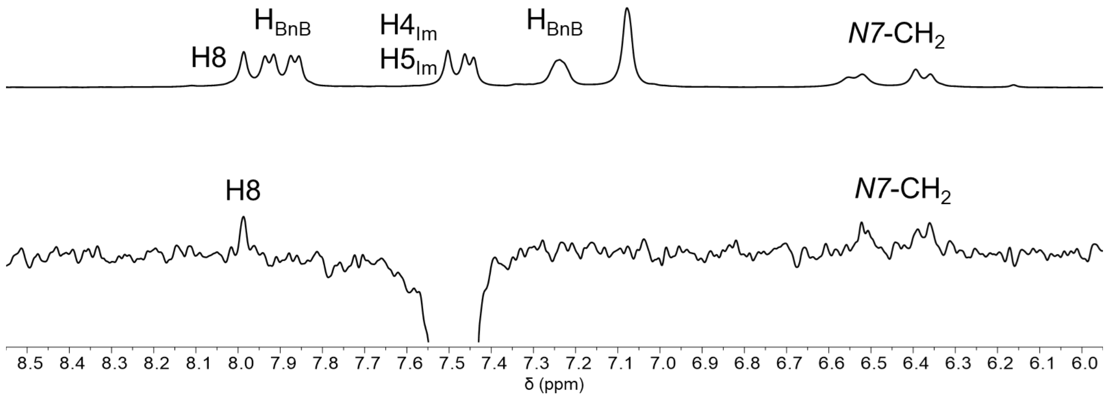

Figure S21. Selected region in the [1]H 1D-selNOE of [S_a-**1**][R_a-BnB] upon irradiation at 7.53 ppm (CD_3CN, 298 K).

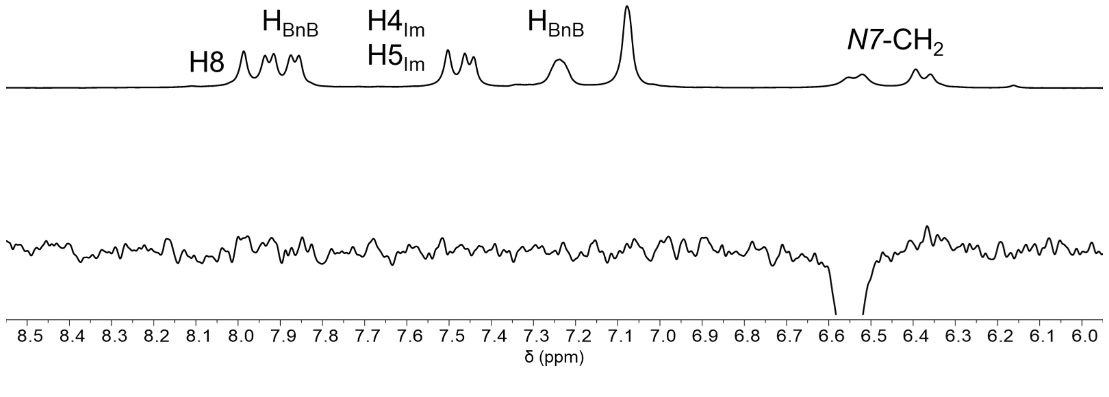

Figure S22. Selected region in the [1]H 1D-selNOE of [S_a-**1**][R_a-BnB] upon irradiation at 6.57 ppm (CD_3CN, 298 K).

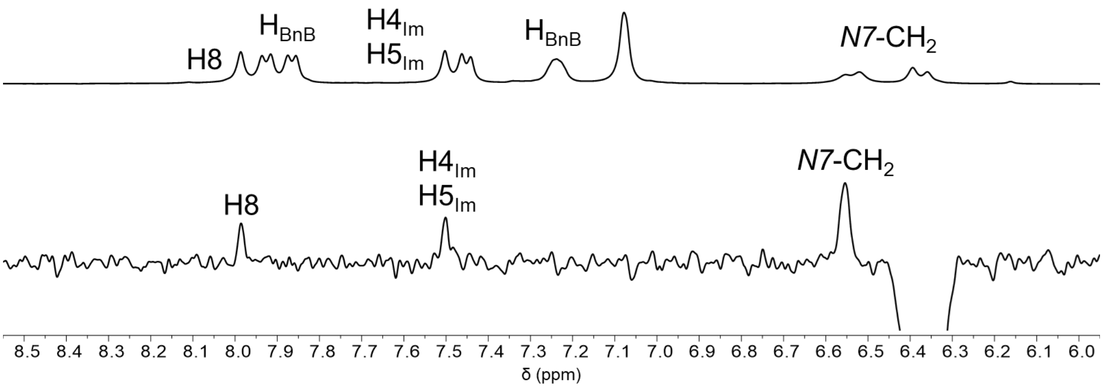

Figure S23. Selected region in the ^{1}H 1D-selNOE of [S_a-**1**][R_a-BnB] upon irradiation at 6.40 ppm (CD_3CN, 298 K).

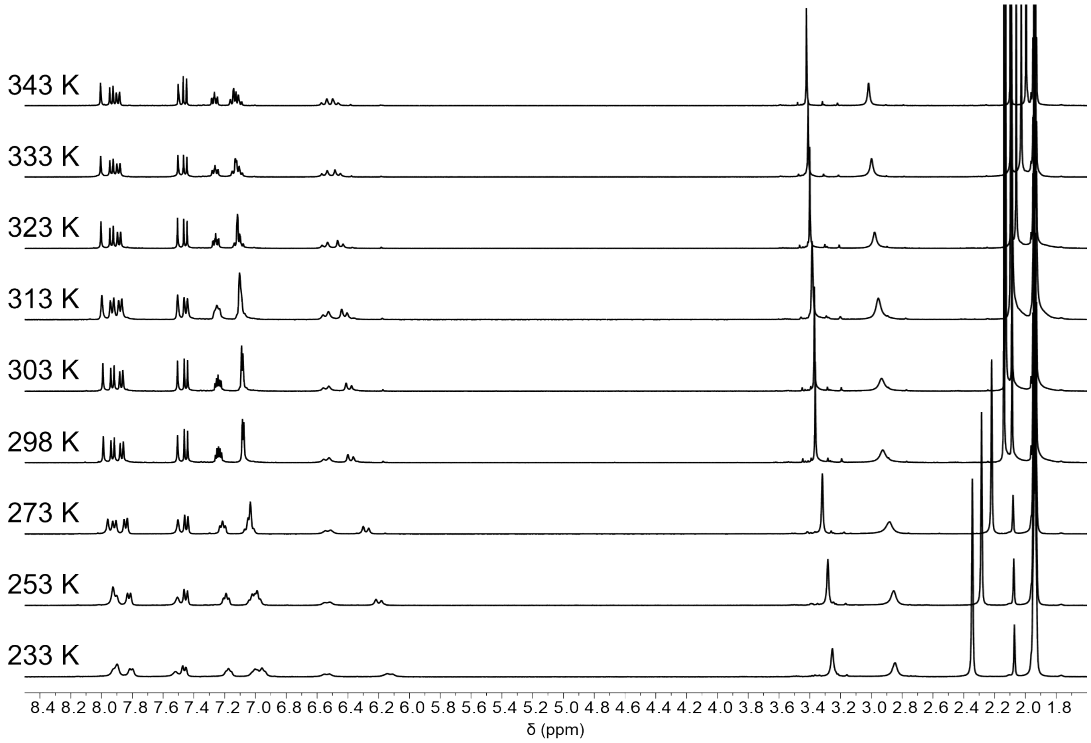

Figure S24. Variable temperature ^{1}H NMR spectra of [S_a-**1**][R_a-BnB] (100 MHz, CD_3CN).

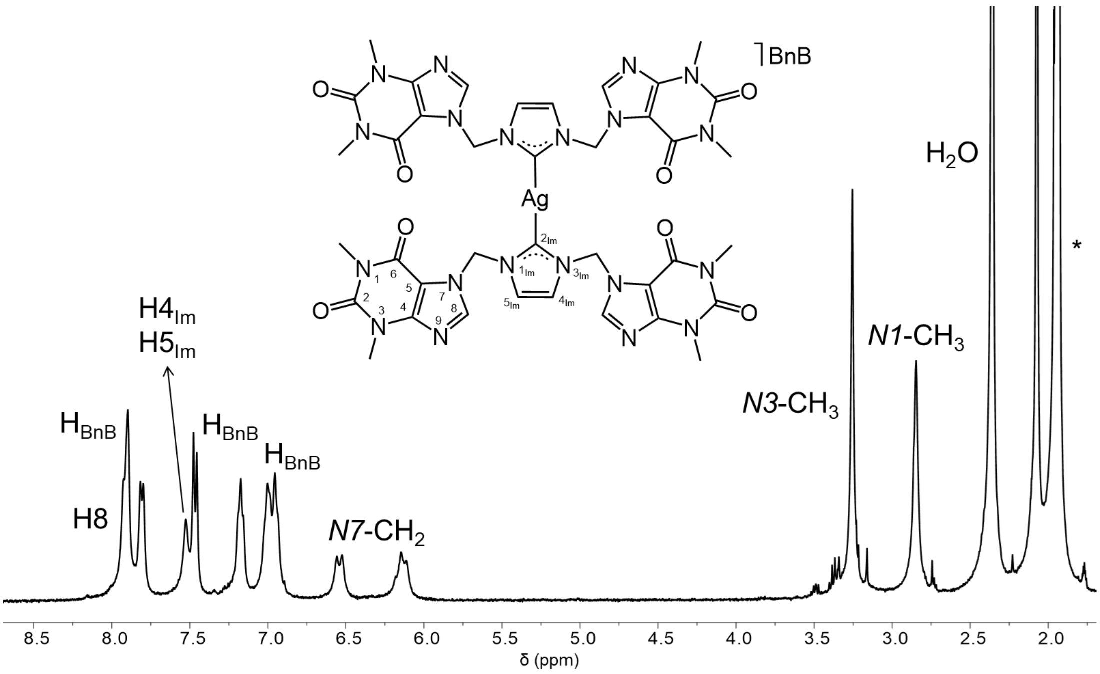

Figure S25. ^{1}H NMR spectrum of [S_a-**1**][R_a-BnB] (400 MHz, CD_3CN, 233 K).

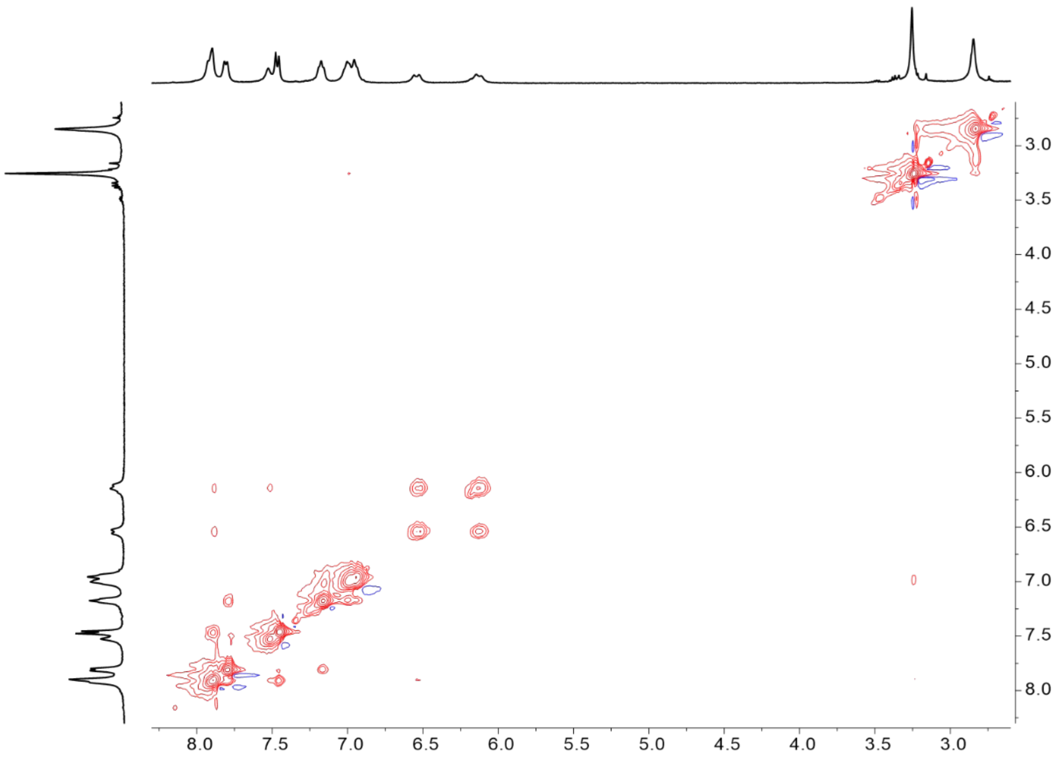

Figure S26. ^{1}H-^{1}H NOESY NMR spectrum of [S_a-**1**][R_a-BnB] (CD_3CN, 233 K).

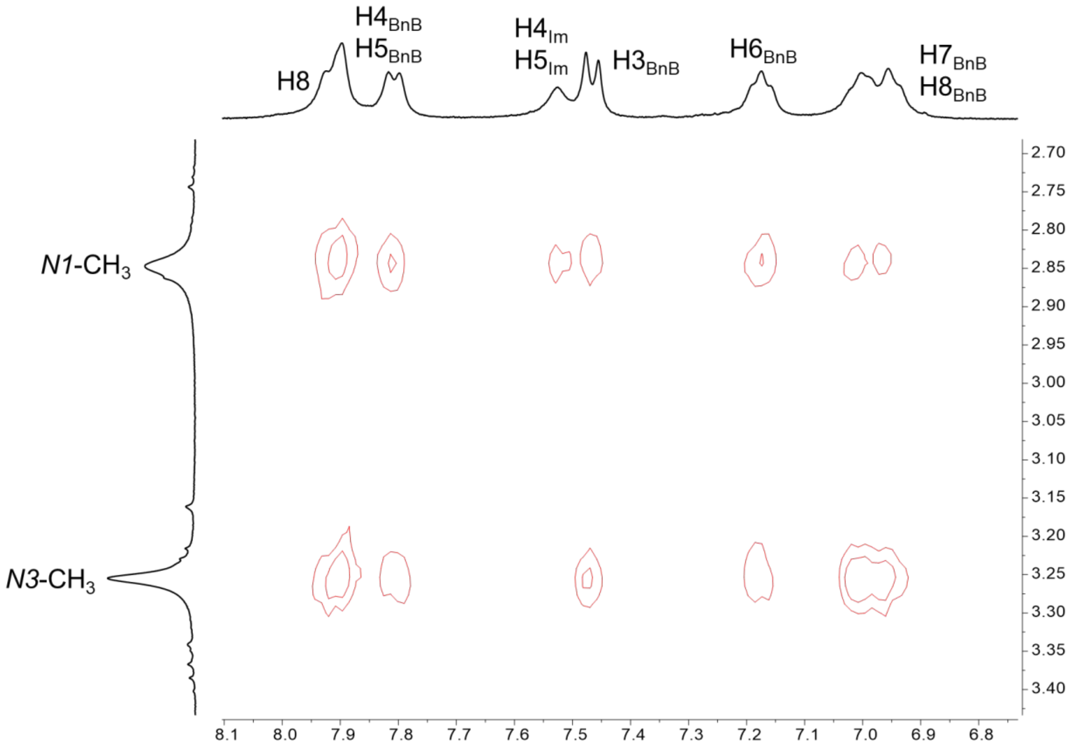

Figure S27. Detail of the ^{1}H-^{1}H NOESY NMR spectrum of [S_a-**1**][R_a-BnB] (CD_3CN, 233 K).

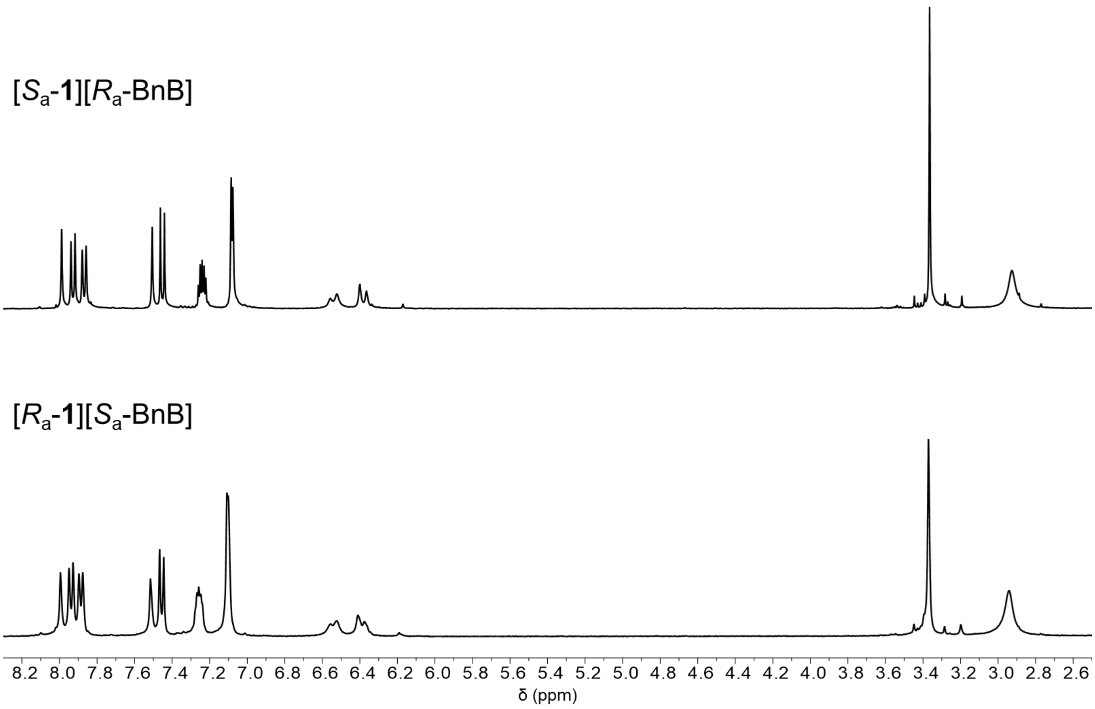

Figure S28. ^{1}H NMR spectra of [S_a-**1**][R_a-BnB] (top) and [R_a-**1**][S_a-BnB] (bottom) (400 MHz, CD_3CN, 298 K).

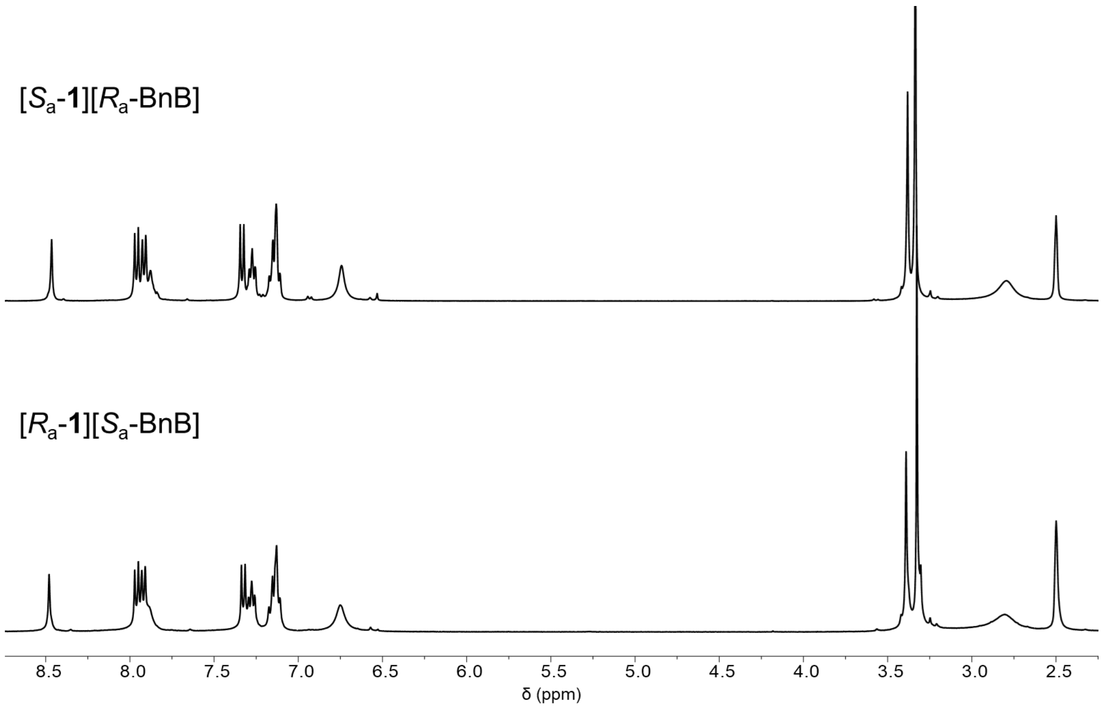

Figure S29. ^{1}H NMR spectra of [S_a-**1**][R_a-BnB] (top) and [R_a-**1**][S_a-BnB] (bottom) (400 MHz, DMSO-d_6, 298 K).

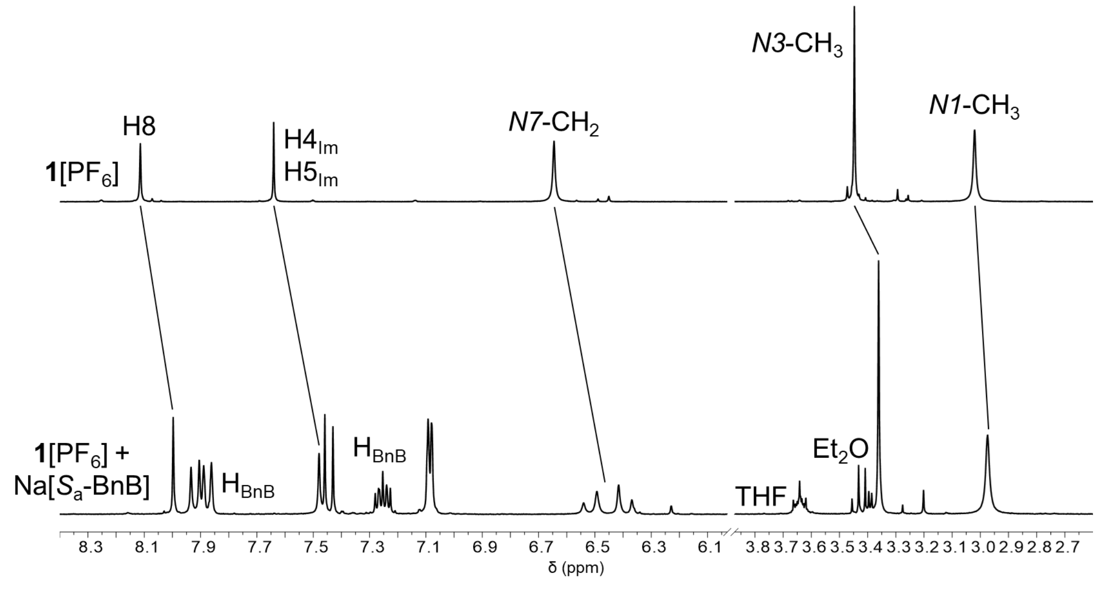

Figure S30. ^{1}H NMR spectra of **1**[PF_6] (top) and [R_a-**1**][S_a-BnB] (bottom) (300 MHz, CD_3CN, 298 K).

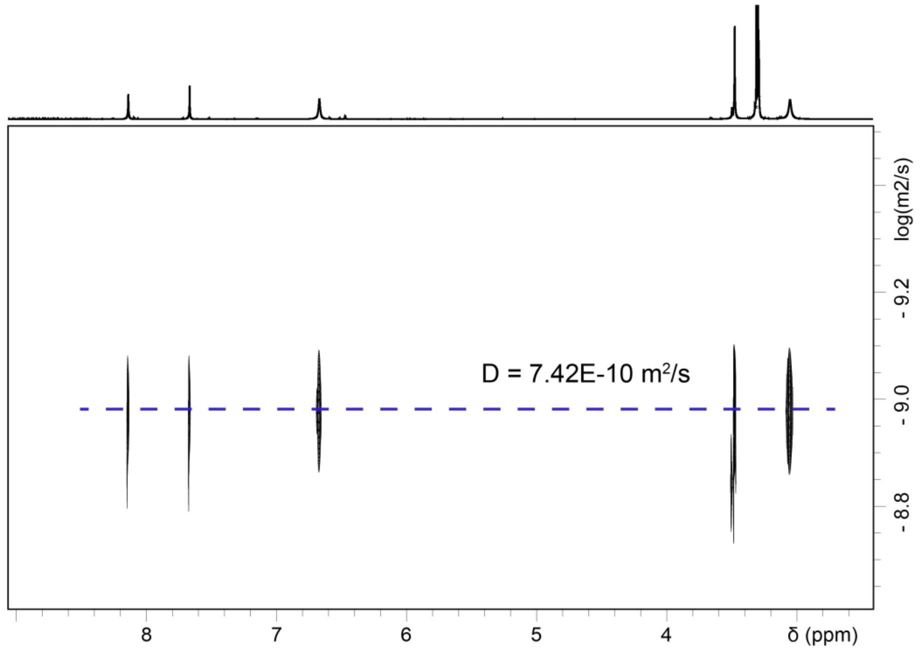

Figure S31. ^{1}H DOSY NMR spectrum of **1**[PF_6] (400 MHz, CD_3CN, 298 K). Diffusion coefficient is highlighted.

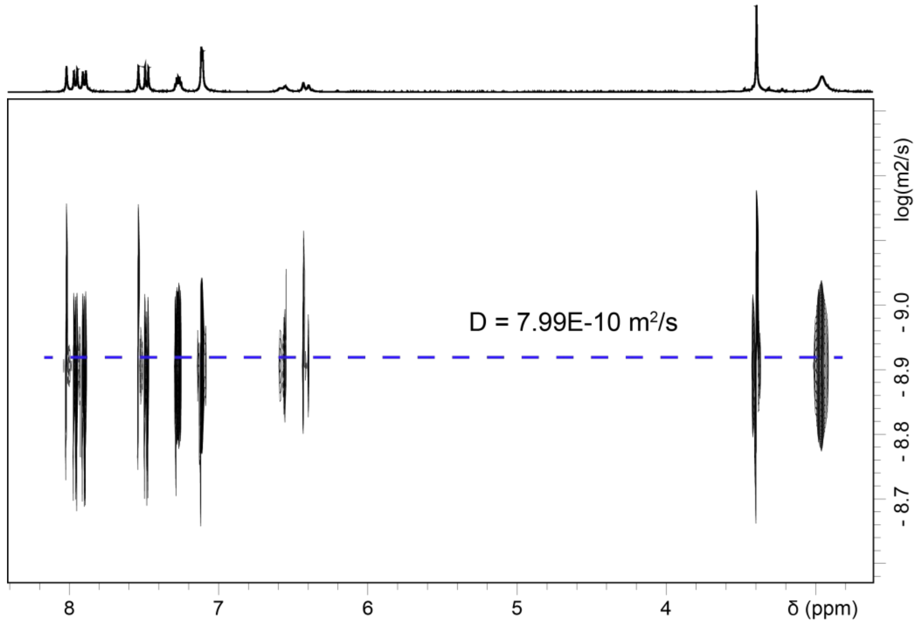

Figure S32. ^{1}H DOSY NMR spectrum of [S_a-**1**][R_a-BnB] (400 MHz, CD_3CN, 298 K). Diffusion coefficient is highlighted.

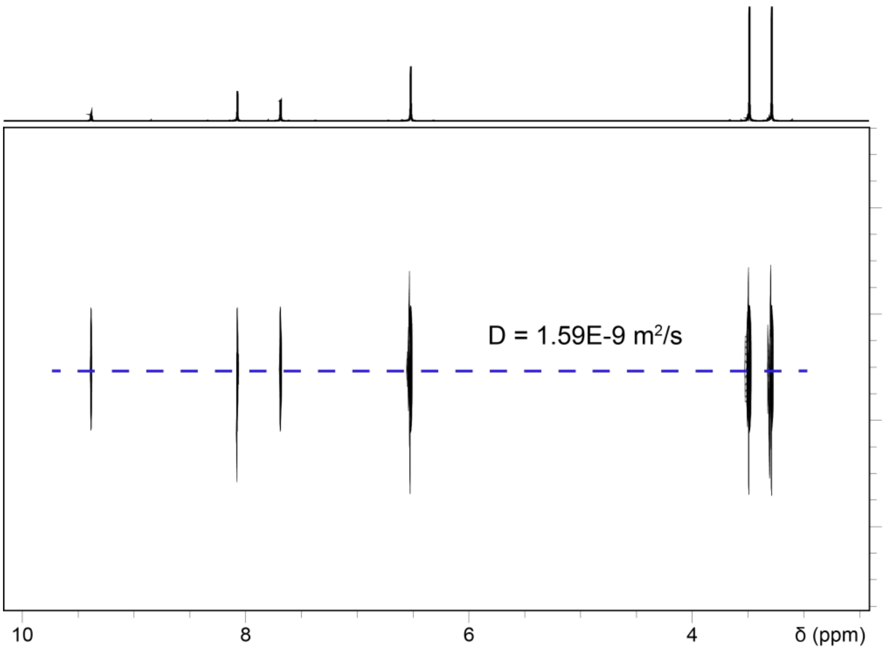

Figure S33. ^{1}H DOSY NMR spectrum of the imidazolium salt precursor [Theo-CH_2-HIm-CH_2-Theo][PF_6], (400 MHz, CD_3CN, 298 K). Diffusion coefficient is highlighted.

HRMS spectra

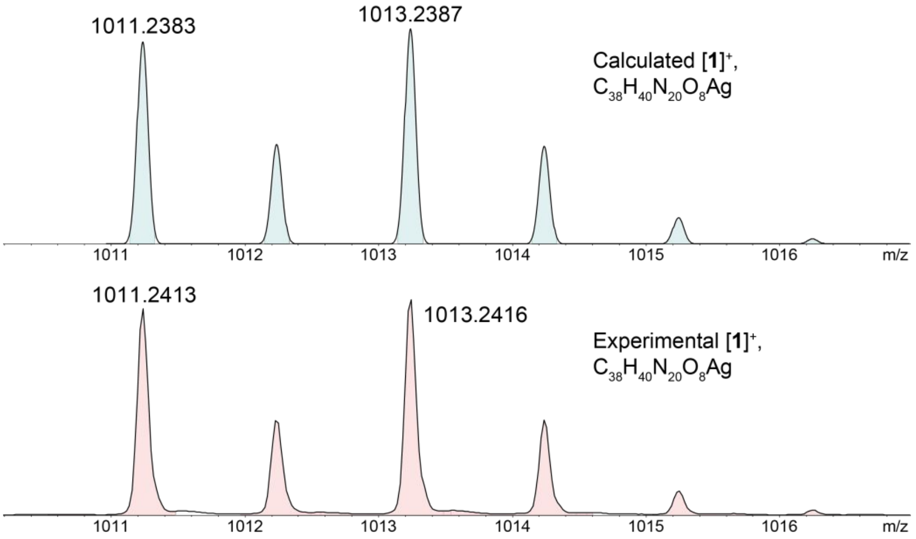

Figure S34. HRMS spectrum of **1**[BnB](ESI+).

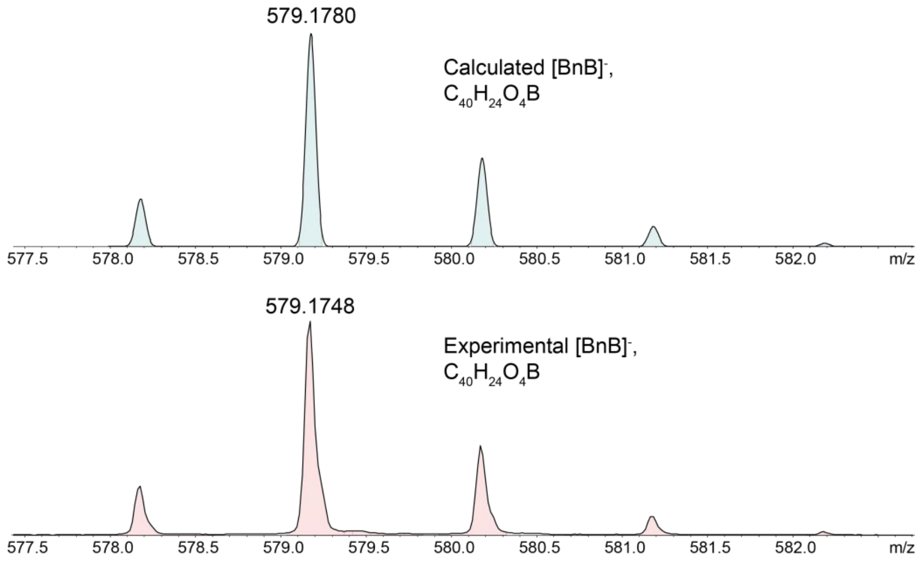

Figure S35. HRMS spectrum of **1**[BnB](ESI–).

UV-VIS spectra

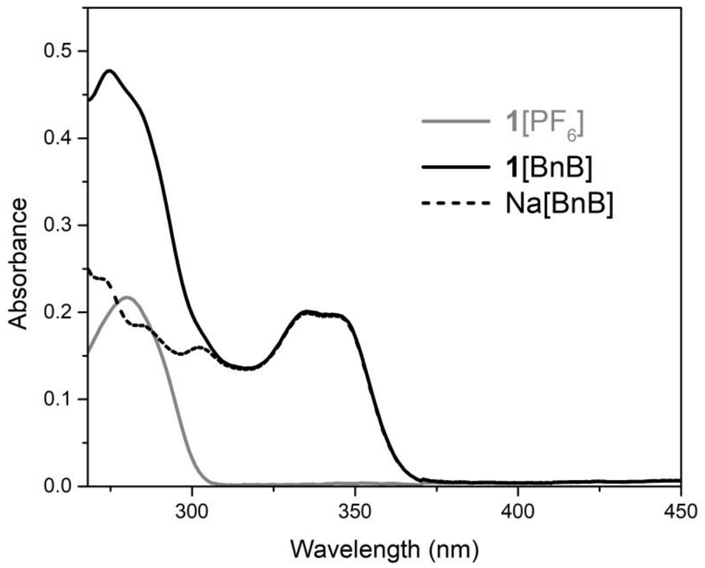

Figure S36. UV-Vis absorption spectra of Na[BnB] and **1**[BnB] in DMSO (10^{-5} M).

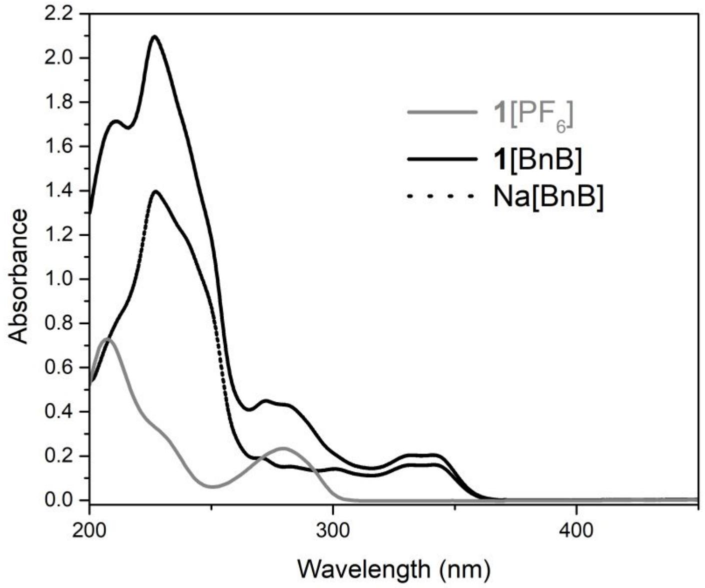

Figure S37. UV-Vis absorption spectra of Na[BnB] and **1**[BnB] in CH_3CN (10^{-5} M).

CD spectra

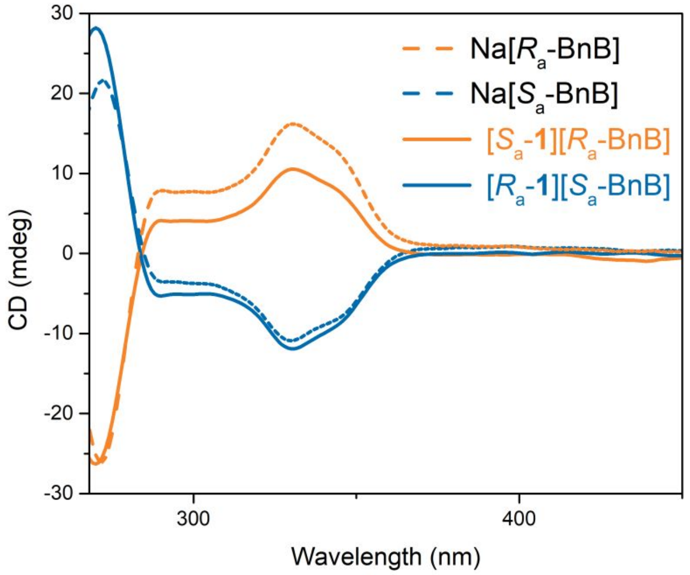

Figure S38. CD spectra of Na[R_a-BnB], Na[S_a-BnB], [S_a-**1**][R_a-BnB] and [R_a-**1**][S_a-BnB] in DMSO (10^{-5} M).

X-ray data

X-ray diffraction data of Na[R_a-BnB]·5THF (CCDC 2477508), [R_a-**1**][S_a-BnB]·DMSO·4EtOAc (CCDC 2477509), and [S_a-**1**][R_a-BnB]·DMSO·4EtOAc (2477510), were collected on a Bruker D8 Venture diffractometer, with graphite-monochromated Mo Kα radiation (λ = 0.71073 Å). Single crystals were mounted and coated with perfluoropolyether oil. Diffracted intensities were integrated with SAINT,[SI-3] and corrected of absorption effects was performed with a multi-scan strategy by SADABS,[SI-4,SI-5] both implemented in the APEX4 software package. Both structures were solved by direct methods with the software SHELXS[SI-6] and refined by full-matrix least squares on F^2 with SHELXL program,[SI-7] and the WinGX system.[SI-8] To refine the crystal structure, the SQUEEZE[SI-9] procedure implemented in PLATON[SI-10] was used to remove the contribution of disordered solvent molecules from the electron density map (see main text for details).

Crystal data for compound Na[R_a-BnB]·5THF: $C_{60}H_{64}BNaO_9$, M_r = 962.91, colorless prism, triclinic *P1*, a = 9.3238(4) Å, b = 11.0708(5) Å, c = 12.7064(6) Å, α = 86.3806(16)°, β = 79.8269(17)°, γ = 77.1408(16)°, V = 1258.19(10) Å^3, Z = 1, T = 100(2) K, D_{calcd} = 1.271 cm^{-3}, μ = 0.091 mm^{-1}, absorption correction factors min. 0.952 max. 0.991, 84412 reflections, 12395 unique (R_{int} = 0.0356), 12067 observed, R_1 = 0.0581 [I > 2σ(I)], wR_2(F^2) = 0.1634 (all data), GOF = 1.040. CCDC 2477508.

Crystal data for compound [R_a-**1**][S_a-BnB]·DMSO·4EtOAc: $C_{96}H_{102}AgBN_{20}O_{21}S$, M_r = 2022.71, colorless block, orthorhombic $P2_12_12_1$, a = 25.9457(15) Å, b = 26.5537(16) Å, c = 29.0480(18) Å, V = 20013(2) Å^3, Z = 8, T = 100(2) K, D_{calcd} = 1.343 cm^{-3}, μ = 0.301 mm^{-1}, absorption correction factors min. 0.893 max. 0.979, 401726 reflections, 49921 unique (R_{int} = 0.1251), 31858 observed, R_1 = 0.0666 [I > 2σ(I)], wR_2(F^2) = 0.1904 (all data), GOF = 1.058. CCDC 2477509.

Crystal data for compound [S_a-**1**][R_a-BnB]·DMSO·4EtOAc: $C_{96}H_{102}AgBN_{20}O_{21}S$, M_r = 2022.71, colorless block, orthorhombic $P2_12_12_1$, a = 25.8948(16)Å, b = 26.6469(17) Å, c = 29.0326(18) Å, V = 20033(2) Å^3, Z = 8, T = 100(2) K, D_{calcd} = 1.341 cm^{-3}, μ = 0.301 mm^{-1}, absorption correction factors min. 0.895 max. 0.977, 1144066 reflections, 49787 unique (R_{int} = 0.1531), 35832 observed, R_1 = 0.0688 [I > 2σ(I)], wR_2(F^2) = 0.1897 (all data), GOF = 1.022. CCDC 2477510.

References

[SI-1] Polo, A.; Gutiérrez Merino, L.; Rodríguez, R.; Sanz Miguel, P. J. Chirality at Metal in a Linear $[Ag(NHC)_2]^+$ Complex: Stereogenic C–Ag–C Axis, Atropisomerism and Role of π-π Interactions. *Chem. Eur. J.* **2024**, *30*, e202403239.

[SI-2] Raskatov, J. A.; Brown, J. M.; Thompson, A. L. Chiral Selection in the Formation of Borates from Racemic Binaphthols and Related Diols. *CrystEngComm* **2011**, *13*, 2923-2929.

[SI-3] Bruker-AXS. SAINT+ Area-Detector Integration Software, version 6.01; Bruker AXS Inc.: Madison, WI, 2001.

[SI-4] Bruker-AXS. SADABS: Area-Detector Absorption Correction Program; Bruker AXS Inc.: Madison, WI, 1996.

[SI-5] Krause, L.; Herbst-Irmer, R.; Sheldrick, G. M.; Stalke, D. Comparison of silver and molybdenum microfocus X-ray sources for single-crystal structure determination. *J. Appl. Crystallogr.* **2015**, *48*, 3-10.

[SI-6] Sheldrick, G. M. A short history of SHELX. *Acta Crystallogr.* **2008**, *A64*, 112-122.

[SI-7] Sheldrick, G. M. Crystal structure refinement with SHELXL. *Acta Crystallogr.* **2015**, *C71*, 3-8.

[SI-8] Farrugia, L. J. WinGX suite for small‐molecule single‐crystal crystallography. *J. Appl. Cryst.* **2012**, *45*, 849-854.

[SI-9] Spek, A. L. *PLATON* SQUEEZE: a tool for the calculation of the disordered solvent contribution to the calculated structure factors. *Acta Cryst.* **2015**, *C71*, 9-18.

[SI-10] Spek, A. L. Single-crystal structure validation with the program *PLATON*. *J. Appl. Cryst.* **2003**, *36*, 7-11.
